# Bioinspired TIMP-1 Nanoparticles Mimic Extracellular Vesicle Anti-angiogenic Activity in Ewing Sarcoma

**DOI:** 10.34133/bmr.0408

**Published:** 2026-09-22

**Authors:** Cristina Liboni, Martina Boaron, Diana Corallo, Sanja Aveic, Marco Ballestri, Ricardo Sánchez-Rodríguez, Alessandra Maria Testa, Claudia Ferroni, Greta Varchi, Antonella Viola, Roberta Angioni

**Affiliations:** ^1^ Laboratory of Immunity and Inflammation, Department of Biomedical Sciences, University of Padova, Padua, Italy.; ^2^ Pediatric Research Institute Città della Speranza Foundation, Padua, Italy.; ^3^ Laboratory of Target Discovery and Biology of Neuroblastoma, Pediatric Research Institute Città della Speranza Foundation, Padua, Italy.; ^4^ Institute for Organic Synthesis and Photoreactivity (ISOF), National Research Council of Italy (CNR), Bologna, Italy.

## Abstract

Anti-angiogenic therapies targeting the vascular endothelial growth factor (VEGF) axis have achieved transient benefits, as tumors rapidly activate compensatory vascular programs. Here, we present a bioinspired nanoplatform that reproduces the anti-angiogenic signaling of mesenchymal-stromal-cell-derived extracellular vesicles (EVs) by delivering the endogenous metalloproteinase inhibitor tissue inhibitor of metalloproteinases-1 (TIMP-1). Poly(methyl methacrylate) nanoparticles (NP_TIMP1) were engineered to retain TIMP-1 bioactivity, inhibit matrix metalloproteinase activity, and efficiently interact with endothelial cells. In vitro, NP_TIMP1 suppressed endothelial sprouting and tip-cell formation in 2-dimensional and 3-dimensional microfluidic systems. In zebrafish patient-derived xenografts of Ewing sarcoma, NP_TIMP1 localized to tumor-associated vessels and substantially reduced vascular infiltration, reproducing the efficacy of TIMP-1-enriched EVs. In contrast, soluble TIMP-1 failed to elicit these effects, indicating that nanoparticle-mediated delivery enhances protein stability and spatial engagement with the endothelium. This study establishes TIMP-1 as a key stromal regulator of angiogenesis and demonstrates that EV-mimetic nanoparticles can recapitulate its therapeutic potential, providing a scalable bioengineering approach to overcome the limitations of natural vesicles and develop next-generation anti-angiogenic nanotherapeutics.

## Introduction

Pathological angiogenesis is a hallmark of tumor progression and therapeutic resistance. Hence, targeting angiogenesis has long been considered a cornerstone in the fight against cancer [1,2]. However, despite the clinical success of vascular endothelial growth factor (VEGF) and VEGF receptor (VEGFR) inhibitors in several malignancies, their benefits remain largely transient due to the high redundancy of angiogenic pathways and the rapid activation of compensatory mechanisms [3]. As a result, tumors frequently develop resistance to therapy, leading to disease progression and poor long-term outcomes. This challenge is particularly evident in rare and aggressive cancers, such as pediatric tumors, including Ewing sarcoma (ES), where the therapeutic window is narrow and conventional treatments are often associated with substantial long-term toxicities [4–6]. These limitations have motivated the search for complementary strategies that target alternative aspects of the angiogenic cascade, ideally acting at levels beyond canonical growth factor signaling.

Recent advances in deciphering the mechanisms underlying intercellular communication have provided unprecedented insights into how cells exchange molecular information within the tumor microenvironment (TME). Of note, extracellular vesicles (EVs) are now recognized as central mediators of intercellular communication within the TME, orchestrating endothelial activation, extracellular matrix (ECM) remodeling, and immune regulation. Importantly, EVs can either promote or suppress angiogenesis within the tumor mass depending on their origin and molecular composition [7]. For instance, tumor-derived EVs have been shown to promote endothelial cell activation and neovascularization, supporting tumor growth and metastasis [8]. Conversely, EVs derived mainly from nonmalignant sources can carry anti-angiogenic (AA) factors and suppress pathological vessel formation [7,9]. Accordingly, we demonstrated that mesenchymal-stromal-cell-derived EVs (MSC-EVs), when exposed to pro-inflammatory priming, acquire potent AA properties both in vitro and in vivo [10]. This dual functionality of EVs has fueled considerable interest in their use as tools to modulate angiogenesis. In particular, the clinical translation of AA EVs has emerged as a promising adjuvant strategy in oncology. However, while EV-based therapies offer a biologically sophisticated means to modulate vascular behavior, their clinical translation remains limited by intrinsic heterogeneity, complex isolation procedures, and poor scalability [11,12]. To overcome these challenges, synthetic nanoparticles (NPs) have been developed to reproduce key EV functions while providing improved control over composition, stability, and manufacturability. The emerging concept of “EV-mimetic” nanoplatforms merges biological insight with materials engineering, enabling precise spatial presentation of bioactive molecules and programmable release kinetics. Such systems can preserve the molecular logic of stromal signaling while overcoming the practical limitations of natural vesicles. Within this framework, we aimed to develop an NP-based therapeutic strategy capable of recapitulating EVs’ AA effects but with improved translational potential.

Here, we report the rational design of polymer-based NPs as a synthetic surrogate of AA EVs. We designed and characterized poly(methyl methacrylate) (PMMA)-based NPs loaded with recombinant tissue inhibitor of metalloproteinases-1 (TIMP-1), an endogenous tissue inhibitor of matrix metalloproteinases (MMPs) that we found playing a major role in the AA activity of MSC-EVs [10]. We demonstrate that NP_TIMP1 retains TIMP-1 bioactivity, efficiently interacts with endothelial cells, and inhibits angiogenic sprouting in advanced in vitro and in vivo models. Using zebrafish patient-derived xenografts of ES, we show that NP_TIMP1 reproduces the efficacy of TIMP-1-enriched EVs in suppressing tumor vascularization, whereas soluble TIMP-1 alone is ineffective. This bioinspired approach establishes a mechanistic and translational bridge between stromal vesicle biology and nanomedicine, paving the way for vesicle-mimetic strategies that integrate biological precision with engineering control.

## Materials and Methods

### Cell culture

Bone-marrow-derived mesenchymal stromal cells (BM-MSCs), isolated and characterized previously as in Zanotti et al. [13], were cultured in 1 g/l Dulbecco’s modified Eagle medium (DMEM) (Lonza, Basel, Switzerland) supplemented with 20% fetal bovine serum (FBS [Labtech, Bergamo, Italy]), 1% l-glutamine (Lonza), and 100 U/ml penicillin/streptomycin (P/S) (Lonza). Confluent cells were subcultured every 2 to 3 d in StemPro Accutase (Gibco, Thermo Fisher Scientific, Milan, Italy). SVEC4-10 cells (American Type Culture Collection [ATCC], Virginia, USA) were cultured in DMEM (ATCC) medium supplemented with 10% FBS (Gibco) and 1% P/S (Lonza). Confluent cells were divided every 2 to 3 d with 2 mM trypsin–EDTA. ES1 cells (obtained from “The Childhood Solid Tumor Network”, St. Jude Hospital, Memphis, TN, USA, under Uniform Biological Material Transfer Agreement) were maintained in DMEM with 4.5 g/l glucose (Lonza) supplemented with 10% FBS (Biowest, Nuaillé, France), 1% l-glutamine (Lonza), and 1% P/S (Lonza) [14]. Confluent cells were subcultured every 2 to 3 d with 2 mM trypsin–EDTA. MCA203 cells (fibrosarcoma cells from C57BL/6 mice H-2b, kindly provided by Prof. Vincenzo Bronte) were cultured in a humidified incubator with 5% CO_2_ at 37 °C, in high-glucose DMEM (Lonza) supplemented with 10% heat-inactivated FBS, 10 mM Hepes (Lonza), and 1 mM P/S (Lonza). Primary human umbilical vein endothelial cells (HUVECs) (ATCC) were cultured in tissue flasks pretreated with 0.8% bovine gelatin in EBM-2 Endothelial Cell Growth Basal Medium-2 (Clonetics, Lonza) supplemented with EGM-2 Endothelial SingleQuots Kit (Clonetics, Lonza) and maintained at 37 °C and 5% CO_2_. Confluent cells were subcultured every 2 to 3 d with StemPro Accutase (Gibco).

### MSC-conditioned medium production

The conditioned medium (CM) used for EV isolation was collected from control or primed murine BM-MSCs, as previously described [10,13]. Briefly, BM-MSCs, cultured as described, were seeded at 50,000 cells/well in complete medium in a 24-well plate. After 24 h, cells were stimulated or not with a priming cocktail (25 ng/ml mIL1β [PeproTech, Thermo Fisher Scientific], 20 ng/ml mIL6 [PeproTech], and 25 ng/ml mTNFα [PeproTech]) for 24 h. Stimuli were removed, and cells were washed 3 times with 1 g/l DMEM (Lonza). Cells were then cultured for 18 h in starvation medium (1 g/l DMEM [Lonza] supplemented with 2 mM glutamine and 100 U/ml P/S). At the end, CMs were harvested, and cellular debris were eliminated by centrifugation (2,000*g*, 10 min) before long-term storage at −80 °C.

### EV isolation and TIMP-1 silencing

EVs were isolated from MSC-CMs (both control and primed) by ultrafiltration as in Angioni et al. [10]. First, Amicon Ultra 15-ml filters (Millipore, Merck KGaA, Darmstadt, Germany) were sterilized in 70% ethanol and then washed twice by centrifuging at 4,000*g* for 10 min. MSC-CMs were then applied to the filters (each filter was loaded with 12 ml of CM, equivalent to a medium conditioned by 1 × 10^6^ cells) and centrifuged at 2,800*g* for 20 min. Filters were washed in 10 ml of phosphate-buffered saline (PBS) before collecting concentrated EVs (approximately 150 μl). EVs were then stored at −80 °C until use. BM-MSCs were silenced for TIMP-1 with a reverse transfection protocol. Briefly, transfection solutions containing 50 μl Opti-MEM and 50 nM small interfering RNA (siRNA) (TIMP-1 [Ambion, Kaufungen, Germany] or silencer negative control [Ambion]) were mixed. On the other side, 1 μl of Lipofectamine 2000 (Thermo Fisher Scientific) was mixed with 50 μl of Opti-MEM. The 2 solutions were incubated separately for 5 min at room temperature (RT) and 15 min together. One hundred microliters of the mixed solution was seeded at the bottom of a 24-well plate, and 50,000 cells MSCs in 400 μl of complete medium were added onto this. Cells were cultured overnight at 37 °C and 5% CO_2_ and then stimulated to harvest CM as described. TIMP-1 silencing in EVs was assessed by Western blot as described.

### Western blot

The presence of selected markers on our preparations was assessed by Western blot, according to the protocol already described by Angioni et al. [10]. Briefly, proteins were extracted from purified EVs with sodium dodecyl sulfate (SDS) 0.4% in PBS. An equal protein amount (about 3 μg, quantified by using the Micro BCA kit [Pierce, Massachusetts, USA]) was separated by 10% SDS–polyacrylamide gel electrophoresis gel under nonreductive conditions (CD63) and reductive conditions (5 mM dithiothreitol [Sigma-Aldrich, Merck], CD73, and TIMP-1). Gels were transferred onto polyvinylidene fluoride membranes, 0.45 μm (Millipore), activated with methanol (Sigma-Aldrich), using Tris-Glycine Transfer Buffer. Following a specific blocking for 1 h (5% bovine serum albumin [BSA; Sigma-Aldrich] in 1× Tris-buffered saline [TBS] with 0.2% Tween 20), primary antibodies (anti-CD63 [MBL, Everswinkel, Germany], anti-CD73 [Abcam, Cambridge, UK], and TIMP-1 [R&D Systems, Minneapolis, USA]), diluted 1:1,000 in 1× TBS, 0.2% Tween, and 1% BSA, were incubated overnight in end-to-end mixing. Then, secondary antibodies were added (ECL anti-rabbit [GE HealthCare, Milan, Italy], ECL anti-rat [GE HealthCare], and anti-goat [Bio-Rad Laboratories, California, USA]), diluted 1:5,000 in 1× TBS, 0.2% Tween, and 1% BSA. The chemiluminescent signal was revealed after membrane incubation with the ECL Prime Western Blot Detection reagent (GE) and imaged with iBright5000 (Thermo Fisher Scientific). Densitometric analysis of protein bands was performed with ImageJ 6.0; data were normalized to the housekeeping protein (CD63) and expressed as fold change.

### EV NP tracking

EV characterization by nanoparticle tracking (NTA) was performed as in Bisaccia et al. [15] using ZetaView PMX220 TWIN (Particle Metrics), plotting the mean size of the particles (as obtained by the instrument).

### NP_TIMP1 synthesis

PMMA-based NPs were synthesized following the procedure described in the literature [16]. In brief, a 100-ml aqueous solution containing 3-sulfopropyl methacrylate potassium salt (0.65 mmol, 151 mg) and SDS (0.01 mmol, 3 mg) was placed in a 250-ml 3-necked flask equipped with mechanical stirring and purged with nitrogen gas. After 15 min, methyl methacrylate (4 ml, 40.2 mmol) was added dropwise, and the mixture was heated to 80 °C. Once this temperature was reached, potassium persulfate (0.25 mmol, 93 mg) was introduced as a radical initiator. The reaction mixture, which formed a white suspension, was maintained under stirring and heating for 4 h and subsequently allowed to cool to RT. The resulting milky dispersion was dialyzed for 5 d against deionized water using Visking tubing (Φ = 28.6 mm, molecular weight cut-off [MWCO] = 12 to 14 kDa), with the external water replaced twice daily. This process yielded a PMMA NP suspension with a final concentration of 17.6 mg/ml. For TIMP-1 loading, 894.32 μl of Milli-Q water and varying volumes (40, 60, and 100 μl) of a TIMP-1 aqueous solution (100 μg/ml) were added to 5.68 μl of the PMMA NP suspension in a glass vial, yielding NP_TIMP1 formulations. Under these conditions, the theoretical loading capacities of the NPs were 4%, 6%, and 10%, respectively.

$$\text{Loading capacity (LC\%)}=\frac{\text{weight of loaded TIMP}-1}{\text{total weight of}\;\mathrm{NPs}}\times 100$$ (1)

The mixture was stirred for 2 h at RT using a digital tube rotator (F205 [Falc Instruments]). Excess salts were then removed via ultrafiltration (Amicon Ultra filters, MWCO = 100 kDa [Millipore]). Dynamic light scattering (DLS) was employed to determine the hydrodynamic diameter and zeta potential at 25 °C using NanoBrook Omni Particle Size Analyzer (Brookhaven Instruments Corporation, New York, NY, USA). The morphology of the NPs was assessed using scanning electron microscopy (SEM) with a Philips XL30 microscope. The samples were sputter-coated under vacuum with a thin layer (10 to 30 Å) of gold.

### EV staining

EVs (either control or primed), at the same concentration, were stained with 1 μM Molecular Probes DiI Stain (1,1′-dioctadecyl-3,3,3′,3′-tetramethylindocarbocyanine perchlorate [Thermo Fisher Scientific]) in end-to-end mixing for 1 h at 4 °C. PBS was used as staining control. EVs were cleaned from excess dye with Pierce Protein Concentrators (MW 3,000) (Thermo Fisher Scientific) according to the manufacturer’s instructions (pre-rinse step in PBS at 12,000*g* for 30 min and concentration step at 12,000*g* for 45 min). DiI-EVs were quantified with Qubit (Thermo Fisher Scientific) to assess protein concentration.

### NP labeling

NPs were fluorescently labeled using rhodamine B isothiocyanate (RBITC [Sigma-Aldrich, Merck Life Science]). RBITC (1.18 μmol) and methyl methacrylate (400 μl; 3.74 mmol) were dissolved in 200 μl of toluene and 50 μl of dimethyl sulfoxide in a 6-ml glass vial, followed by brief sonication to ensure complete solubilization. The resulting solution was added dropwise at 0 °C to a beaker containing potassium persulfate (53.4 μmol), sodium 3-mercapto-1-propane sulfonate (49.1 μmol), and SDS (40.5 μmol) dissolved in 10 ml of Milli-Q water. The mixture was sonicated using an ultrasonic homogenizer (UP400st, 20% amplitude, 39 W) for 30 min over 4 cycles until a homogeneous emulsion was obtained. The emulsion was then transferred to a 25-ml 3-necked flask, purged with nitrogen for 3 min, and heated to 80 °C under stirring for 4 h to initiate polymerization. The progress of the reaction was monitored by DLS and *ζ*-potential measurements. After completion, the reaction mixture was purified by dialysis (MWCO 12 to 14 kDa) against 400 ml of Milli-Q water until the external water conductivity remained constant. The resulting light pink NP dispersion was further purified using Amicon ultrafiltration units (MWCO 100 kDa) with 500 ml of Milli-Q water. The final NP suspension had a concentration of approximately 10.04 mg/ml, as determined by using the Hetovac VR-1 vacuum heated centrifuge. The NPs exhibited an average diameter of 140 nm and a zeta potential of −26.82 mV. The overall yield of the reaction was 51.2%.

### DiI-EV and NP uptake

For the DiI-EV uptake assay, 100,000 SVEC4-10 cells were seeded the day before into a 48-well plate (Falcon, Merck). The following day, cells were treated with 1.75 μg/ml DiI-stained EVs (see the staining protocol) in the presence or not of murine 50 ng/ml VEGF (PeproTech) in DMEM (ATCC) + 1% P/S. After 6 or 24 h, respectively, cells were detached in StemPro Accutase (Gibco) and centrifuged at 400*g* for 5 min. DiI-positive cells were assessed by using FACS Celesta (BD, New Jersey, USA). At least 2 replicates per condition were prepared. Data were analyzed with the FlowJo 10.0 software (Tree Star Inc., Oregon, USA) gating the number of cells positive for DiI within singlets.

Similarly, for the NP uptake assay 30,000 HUVECs were seeded into each well of a 48-well plate, precoated with 0.8% gelatin in 1× PBS. The following day, one experimental group was treated with only labeled NP_TIMP1, while the other was treated with both labeled NP_TIMP1 and 50 ng/ml VEGF (Sigma-Aldrich, Merck Life Science). Control cells were treated with either PBS or 50 ng/ml VEGF. Twenty-four hours later, cells were washed in PBS, detached with StemPro Accutase (Thermo Fisher Scientific), spun down for 2 min at 2,000 RPM, at RT, and resuspended in 150 μl of FACS buffer (FBS diluted at 2% in PBS, no calcium, no magnesium). NPs’ fluorescence was detected by using FACS Celesta (BD), and data were analyzed using the FlowJo 10.0 software (TreeStar), to quantify the percentage of positive cells over the total cell number and mean fluorescence intensity.

### Fibrin bead assay

Fibrin bead assay (FBA) was performed modifying the protocol already published by Carpentier et al. [17]. A total of 0.05 g of dry Cytodex 3 microcarrier beads (Sigma-Aldrich, Merck Life Science) was hydrated in 5 ml of 1× PBS for at least 3 h in a rocker at a concentration of 30,000 beads/ml. They were left to settle down for 15 min and then were autoclaved for 20 min at 121 °C. Ready-to-use beads were stored at 4 °C. The number of beads required was 80 beads per well, which were washed in warm medium (DMEM [ATCC] + 2% FBS [Gibco]) at a concentration of 2,500 beads/ml on the day of the experiment. They were then let to settle for 30 min at RT. SVEC4-10 cells were mixed with beads at a concentration of 400 cells/beads for 4 h at 37 °C, inverting the suspension every 20 min. Coated beads were incubated at 37 °C and 5% CO_2_ overnight in a 25-cm^2^ flask with 5 ml of medium. The following day, beads were collected and transferred to a 15-ml tube where they were let to settle down for 30 min and then washed twice in PBS. Beads were then resuspended at a concentration of 1,000 beads/ml in a solution of 2.5 mg/ml fibrinogen (Sigma-Aldrich, Merck Life Science) dissolved in water bath 37 °C in PBS, sterile-filtered, and supplemented with 0.15 U/ml aprotinin (Sigma-Aldrich, Merck Life Science). A total of 4 μl of 10 U/ml thrombin (Sigma-Aldrich, Merck Life Science) was added at the bottom of each well in a flat-bottom 96-well plate; 80 μl of bead solution and 80 μl of stimuli were added to each well and gently mixed with thrombin to let the solution solidify. Stimuli were constituted of either 100 ng/ml murine vascular endothelial growth factor (mVEGF) (PeproTech) in cell medium, 100 ng/ml mVEGF (PeproTech) + 2 μM/5 μM MMP inhibitor (Selleck Chemicals, Texas, USA), or 100 ng/ml mVEGF (PeproTech) + 32.85 μl/ml pEVs in cell medium. The plate was left for 5 min at RT to solidify and then incubated for 3 d at 37 °C and 5% CO_2_. Sprouting was then imaged with an Axio Observer inverted microscope with incubation (Zeiss, Oberkochen, Germany) and an Axiocam 702 mono camera (Zeiss). Images in bright field were acquired with the ZEN blue 3.3 software (Zeiss), at 10× magnification. The percentage of area occupied by the sprouting was quantified with ImageJ, after defining a region of interest and fixing a threshold for signal positivity, with results expressed as arbitrary units (a.u.), normalized over the experimental group not treated with VEGF and pEVs.

### MMP zymography

The ability of NP_TIMP1 and recombinant human TIMP-1 (rhTIMP1) to suppress MMP activity was analyzed with an MMP zymography assay; 0.1 μM MMP inhibitor (Selleck Chemicals), 200 ng/ml rhTIMP1 (Bio-Techne, Minneapolis, USA), or increasing amounts of NPs (75/125/250/500 ng) loaded with 10 μg/ml rhTIMP1 were diluted in MMP activity buffer (Millipore). Then, each sample was supplemented with 0.01 μM active MMP9 (Sigma-Aldrich) and was incubated for 10 min. MMP Green Substrate (Abcam) was added to each sample and incubated for 10 min before kinetic reading. Finally, fluorescence (490 nm ex/525 nm em) was measured with Spark Microplate Reader (Tecan Italia Srl, Milan, Italy) for 15 min. The percentage of enzymatic activity was calculated using the Lineweaver–Burk plot.

### Matrigel tube formation assay

Matrigel Basement Membrane Matrix (Corning Life Sciences, Massachusetts, USA) was thawed overnight at 4 °C. Flat-bottom 96-well plates were coated with 80 μl of Matrigel and incubated at 37 °C and 5% CO_2_ for 30 min to allow polymerization. A total of 20,000 HUVECs for each well were resuspended in 100 μl of assay medium (EBM-2 Basal Medium, 10% FBS), containing either 10 μl of NPs (0.1 mg/ml) loaded with different concentrations of hTIMP-1 (2/3/5 μg) or 10 μl of pEVs (32.85 μl/ml), and seeded drop by drop onto a single well. Each treatment was performed in technical duplicates. For all experiments, seeded cells were incubated for 6 h at 37 °C and 5% CO_2_ to allow the formation of sprouting. Live images from at least 3 random areas of each well were acquired with a phase-contrast inverted microscope, using a 4× objective. Image analysis was performed using the “Angiogenesis Analyzer” plug-in in ImageJ, quantifying both the total tube length and the number of nodes.

### Three-dimensional angiogenesis on-a-chip

OrganoReady Collagen 3-lane 64 (Mimetas, Oegstgeest, the Netherlands) is a microfluidic platform containing 64 independent tissue culture chips, with each chip housing a collagen-loaded central channel and perfusion channels on each side for cell culturing. HUVEC cultures were established following the manufacturer’s instructions. Briefly, HUVECs were seeded into the right channel of every chip at a cell density of 15,000 cells/chip and left at a 75° angle on the plate stand for 2 h to allow cells to adhere to the collagen matrix. Cells were cultured with 100 μl of cell culture medium for 3 d at 37 °C and 5% CO_2_, until the complete formation of a tubular structure. To allow a constant flow of cell medium through the microfluidic chip, the OrganoReady plate was placed on OrganoFlow (Mimetas), a rocking platform that allowed culturing at an inclination of 14° and 8-min intervals. Following the provided protocol, 100 μl of angiogenic stimulus was added on the left channel of every chip, 3 d after seeding. The angiogenic cocktail (“Angio cocktail”) was composed of recombinant human VEGF (Sigma-Aldrich, Merck Life Science), recombinant human fibroblast growth factor 2 (FGF-2) (Sigma-Aldrich, Merck Life Science), recombinant human monocyte chemoattractant protein-1 (MCP-1) (Sigma-Aldrich, Merck Life Science), recombinant human hepatocyte growth factor (HGF) (Sigma-Aldrich, Merck Life Science), phorbol-12-myristate (Sigma-Aldrich, Merck Life Science), and sphingosine 1-phosphate (Sigma-Aldrich, Merck Life Science). For each chip, either 10 μl of NP_TIMP1 (0.1 mg/ml, with 10 μg/ml of rhTIMP1), 10 μg/ml of rhTIMP1 (1×), or 100 μg/ml of rhTIMP1 (10×) in 100 μl of culture medium was perfused on the right channel, in direct contact with cells.

Three days post-treatment, live imaging was performed with an Axio Observer inverted microscope with incubation (Zeiss) and an Axiocam 702 mono camera (Zeiss). Images in bright field were acquired with the ZEN blue 3.3 software (Zeiss), at 5× magnification. The level of sprouting was quantified with ImageJ, after defining a region of interest and fixing a threshold for signal positivity, with results expressed as arbitrary units (a.u.), normalized over the experimental group not treated with NP_TIMP1.

### Immunofluorescence on-a-chip

Immunofluorescence was performed directly on 3-dimensional (3D) HUVEC cultures in an OrganoReady Collagen 3-lane 64 plate (Mimetas) following the provided protocol. Briefly, chips were fixed with 4% paraformaldehyde for 15 min, washed for 5 min with a washing solution (4% FBS in PBS), permeabilized with a permeabilization buffer (0.3% Triton X-100 in PBS) for 5 min, and blocked with a blocking solution (2% FBS, 2% BSA, and 0.1% Tween 20 in PBS) for 45 min; 3D cultures were then incubated with endothelial-cell-specific molecule 1 (ESM1) primary antibody (R&D) on the OrganoFlow (Mimetas) rocker platform at a 5° angle with a 3-min intervals at RT for 2 h. After 2 washes of 3 min each, cells were incubated with the Alexa Fluor 647 chicken anti-goat IgG secondary antibody (Thermo Fisher Scientific) and Texas Red phalloidin (Thermo Fisher Scientific) on the rocker for 30 min at RT. After another 2 washes, cells were stained with Hoechst 33342, trihydrochloride trihydrate (Thermo Fisher Scientific), on the rocker for 15 min at RT. A final 5-min wash with PBS was performed. Images were acquired with an Axio Observer inverted microscope with incubation (Zeiss) and an Axiocam 702 mono camera (Zeiss). Images in bright field were acquired with the ZEN blue 3.3 software (Zeiss), at 20× magnification. The percentage of the ESM1^+^ area was quantified using ImageJ, fixing a threshold for signal positivity, normalized over the experimental group not treated with NP_TIMP1.

### Bulk RNA-seq

Bulk RNA sequencing (RNA-seq) was performed from 3D HUVEC cultures in an OrganoReady Collagen 3-lane 64 plate (Mimetas). HUVEC cultures were established following the manufacturer’s instructions, seeding the cells in the right channel of every chip at a density of 15,000 cells/chip. Cells were cultured with 100 μl of cell culture medium for 3 d at 37 °C and 5% CO_2_, until the complete formation of a tubular structure. Following the angiogenesis protocol, a total of 100 μl of angiogenic cocktail (VEGF, FGF-2, MCP-1, HGF, phorbol-12-myristate, and sphingosine 1-phosphate) was added on the left channel of every chip 3 d after HUVEC seeding. For each chip, 100 μl of culture medium was perfused on the right channel in the presence or not of 10 μl of NP_TIMP1 (0.1 mg/ml, with 10 μg/ml of rhTIMP1). Total RNA was extracted 3 d after treatment following the provided protocol. Briefly, 50 μl of RLT lysis buffer (Qiagen) supplemented with 40 mM dithiothreitol was added in each chip. The buffer was incubated for 60 s, and the lysate from 5 or 6 chips of the same condition was pooled in an RNase-free Eppendorf tube, to reach an adequate final RNA concentration. Total RNA was isolated using the RNeasy Mini Kit (Qiagen) according to the manufacturer’s instructions. The extracted RNA was quantified fluorometrically using Qubit 4.0 Fluorometer (Invitrogen, Carlsbad, CA, USA), while RNA integrity was assessed using an Agilent TapeStation system (Agilent Technologies, Santa Clara, CA, USA). Libraries were prepared using the VAHTS Universal V10 RNA-Seq Library Preparation Kit together with the Ribo-off rRNA Depletion Kit (Vazyme, Nanjing, China), following the manufacturer’s instructions. Libraries were sequenced in paired-end 150-bp mode on a NovaSeq X Plus platform (Illumina, San Diego, CA, USA). Quality control of raw sequencing reads was performed using FastQC (Babraham Bioinformatics). Reads were aligned to the human GRCh38 reference genome using STAR and a reference based on GENCODE v32 gene annotations (Ensembl release 98), corresponding to the 10× Genomics reference package refdata-cellranger-arc-GRCh38-2020-A-2.0.0. Gene-level read counts were generated using HTSeq, integrated within the STAR workflow. Differential expression analysis was performed using the edgeR package (v3.8.2). Briefly, low-expressed genes were filtered out, retaining genes with counts per million (CPM) > 2 in at least 2 samples. Raw counts were normalized using the trimmed mean of M-values method implemented in the calcNormFactors function from edgeR. Differential expression testing was performed using the glmLRT function with the appropriate contrast specification. Multiple testing correction was carried out using the Benjamini–Hochberg procedure, and genes with a false discovery rate <0.1 were considered differentially expressed genes (DEGs). Functional enrichment analysis of DEGs was performed using the clusterProfiler package (v4.18.4). Data visualization was carried out using ggplot2 (v4.0.2), while heatmaps were generated with Heatmap function implemented in the ComplexHeatmap package (v2.26.1) using normalized expression values (logCPM).

### Proteome profiler array

Angiogenesis-associated proteins in treated HUVECs were analyzed using the Proteome Profiler Human Angiogenesis Array Kit (Bio-Techne, R&D Systems, USA), which enables the simultaneous semiquantitative detection of 55 angiogenesis-associated proteins on nitrocellulose membranes spotted with duplicate capture antibodies. Briefly, HUVECs were seeded into a 6-well plate at a concentration of 150,000 cells/well; after 24 h, they were treated with either 50 ng/ml recombinant human vascular endothelial growth factor (hVEGF) or 50 ng/ml hVEGF + 100 μl of NP_TIMP1 in EBM-2 Endothelial Cell Growth Basal Medium supplemented with only FBS and P/S. After 24 h, cells were solubilized in lysis buffer (1% NP40 CA-630, 20 mM tris–HCl pH 8.0) for 30 min at 4 °C and then centrifuged; the supernatant was retrieved and stored at −80 °C. The array was performed following the manufacturer’s instructions: briefly, samples were incubated with a biotinylated detection antibody cocktail and applied to the membranes overnight at 4 °C. After washing, membranes were incubated with streptavidin–horseradish peroxidase (HRP) and developed using ECL Prime Western Blotting Detection Reagent (Cytiva, Danaher Corporation, USA). Chemoluminescent signal intensity was detected by UVITEC HD9 (Eppendorf) and quantified by densitometric analysis using ImageJ, with spot pixel densities normalized against background and compared between samples to determine relative protein expression levels.

### Time-course analysis of TIMP-1 retention on NP_TIMP1

In a thermostated chamber at 37 °C, 1 ml of an aqueous NP_TIMP1 solution containing 0.1 mg of NPs loaded with 10 μg of protein was placed into a dialysis filter (Spectra/Por Float-A-Lyzer G2 Dialysis Device, MWCO 300 kDa). The filter was then immersed in a 100-ml beaker containing 60 ml of PBS under gentle magnetic stirring. Aliquots of 100 μl were collected at 0, 1, 2, 3, 4, 5, 6, and 7 h and subsequently at 24, 48, and 72 h. After each sampling, an equal volume of fresh PBS was added to maintain a constant release medium volume.

Human recombinant TIMP-1 (rhTIMP1) levels in these samples were quantified using the ELISA DuoSet Development System (R&D Systems) according to the manufacturer’s instructions. Briefly, a 96-well plate was coated overnight at RT with the capture antibody, washed, and blocked with the block buffer (1% BSA in PBS) for 1 h. Samples and standards were added in duplicate and incubated for 2 h at RT, followed by incubation with detection antibody for 2 h and streptavidin–HRP for 20 min. After washing steps, 3,3′,5,5′-tetramethylbenzidine (TMB) substrate solution was added for 20 min in the dark, and the reaction was stopped with the enzyme-linked immunosorbent assay (ELISA) stop solution (methanesulfonic acid). Optical density was measured at 450 nm with wavelength correction at 570 nm by using the Infinite M200PRO (Tecan) instrument. Concentrations were calculated from a 7-point standard curve generated in GraphPad Prism 10.

### Zebrafish embryo handling and in vivo EV biodistribution

Following fertilization, embryos were maintained at 28.5 °C in zebrafish egg water supplemented with 0.003% phenylthiourea. All experimental procedures were approved by the Italian regulatory authorities for animal research. Twenty nanoliters of DiI-EVs (32.5 μg/ml) (0.65 ng EVs/embryo) was injected into the duct of Cuvier of 48 h postfertilization (hpf) *Tg(KDRL:GFP)* embryos [18] using a pneumatic picopump (World Precision Instruments, USA) and borosilicate glass capillary needles (outer diameter/inner diameter: 1.00/0.75 mm). At 24 h postinjection (hpi), embryos were anesthetized with 0.003% tricaine and embedded in 1.5% low-melting-point agarose (Sigma-Aldrich, Merck Life Science) in lateral orientation for imaging with an LSM800 confocal microscope (Zeiss).

### In vivo angiogenesis model with EVs

The zebrafish xenograft model employed for the in vivo assessment of angiogenesis was adapted with minor modifications from Nicoli and Presta [19]. Briefly, cells (MCA203 or ES1) were prelabeled with Hoechst 33342 (Thermo Fisher Scientific) and resuspended in Matrigel (Corning) at a concentration of 95,283 cells/ml. EVs were added to the cell suspension at a 3:7 ratio, corresponding to an injection dose of approximately 333.33 cells and 165 pg of EVs per embryo. Transgenic *Tg(KDRL:GFP)* embryos at 48 hpf were injected into the duct of Cuvier with 5 nl of a cell suspension (MCA203 or ES1) combined with EVs.

### In vivo NP biodistribution

Ten nanoliters of alternatively PBS, NP_TIMP1 (10 μg/ml), or NP_empty (10 μg/ml) (100 pg of NPs/embryo) was injected into the duct of Cuvier of 48-hpf *Tg(KDRL:GFP)* embryos [18] using a pneumatic picopump (World Precision Instruments, USA) and borosilicate glass capillary needles (outer diameter/inner diameter: 1.00/0.75 mm). At 24 hpi, embryos were anesthetized with 0.003% tricaine and embedded in 1.5% low-melting-point agarose (Sigma-Aldrich, Merck Life Science) in lateral orientation for imaging with an LSM800 confocal microscope (Zeiss).

### In vivo angiogenesis model with NPs and rhTIMP1

The zebrafish xenograft model employed for the in vivo assessment of angiogenesis was adapted with minor modifications from Nicoli and Presta [19]. Briefly, 1 × 10^6^ ES1 or MCA203 cells were prelabeled with either Hoechst 33342 (Thermo Fisher Scientific) or Qdot 655 ITK Carboxyl Quantum Dots (Thermo Fisher Scientific), in accordance with the manufacturer’s instructions, and resuspended in 10 μl of a solution composed as following: 75% Matrigel (Corning) and 25% of alternatively PBS (negative control), fluorescently labeled NP_TIMP1 (10 μg/ml), fluorescently labeled NP_empty (10 μg/ml), or rhTIMP1 (10 μg/ml). Transgenic *Tg(KDRL:GFP)* embryos at 48 hpf were injected into the duct of Cuvier with 5 nl of the cell suspension combined with PBS, NP_TIMP1, NP_empty, or rhTIMP1. This corresponds to an injection dose of approximately 500 cells and 50 pg of NPs per embryo.

### Statistical analysis

Statistical analysis was performed in Excel 6.0 and GraphPad Prism 9.0. The Gaussian distribution of data was assessed by Shapiro–Wilk and D’Agostino–Pearson omnibus normality tests. According to normal or nonnormal data distribution, a *t* test or a Mann–Whitney test was applied (2-group comparison). For 3 or more groups’ comparison, one-way analysis of variance (normal distribution) or a Kruskal–Wallis test with Dunn’s correction (nonnormal distribution) was applied. At least 2 independent experiments per dataset were performed. The exact number of replicates/embryos is indicated in the figure legend. For zebrafish in vivo experiments, each dot in a graph represents one fish. The number of fish per group is stated in the figure legend.

## Results

### AA pEVs target the endothelium in vivo

Murine MSC-EVs, previously primed with pro-inflammatory cytokines (hereafter referred to as pEVs), have been shown by our group to possess robust AA activity both in vitro and in vivo [10]. However, their biodistribution and uptake dynamics in physiopathological settings remain poorly characterized, limiting both mechanistic insight and translational potential. Hence, we first sought to rigorously assess their in vivo distribution and endothelial tropism and revalidate their functional activity.

**Fig. 1.**
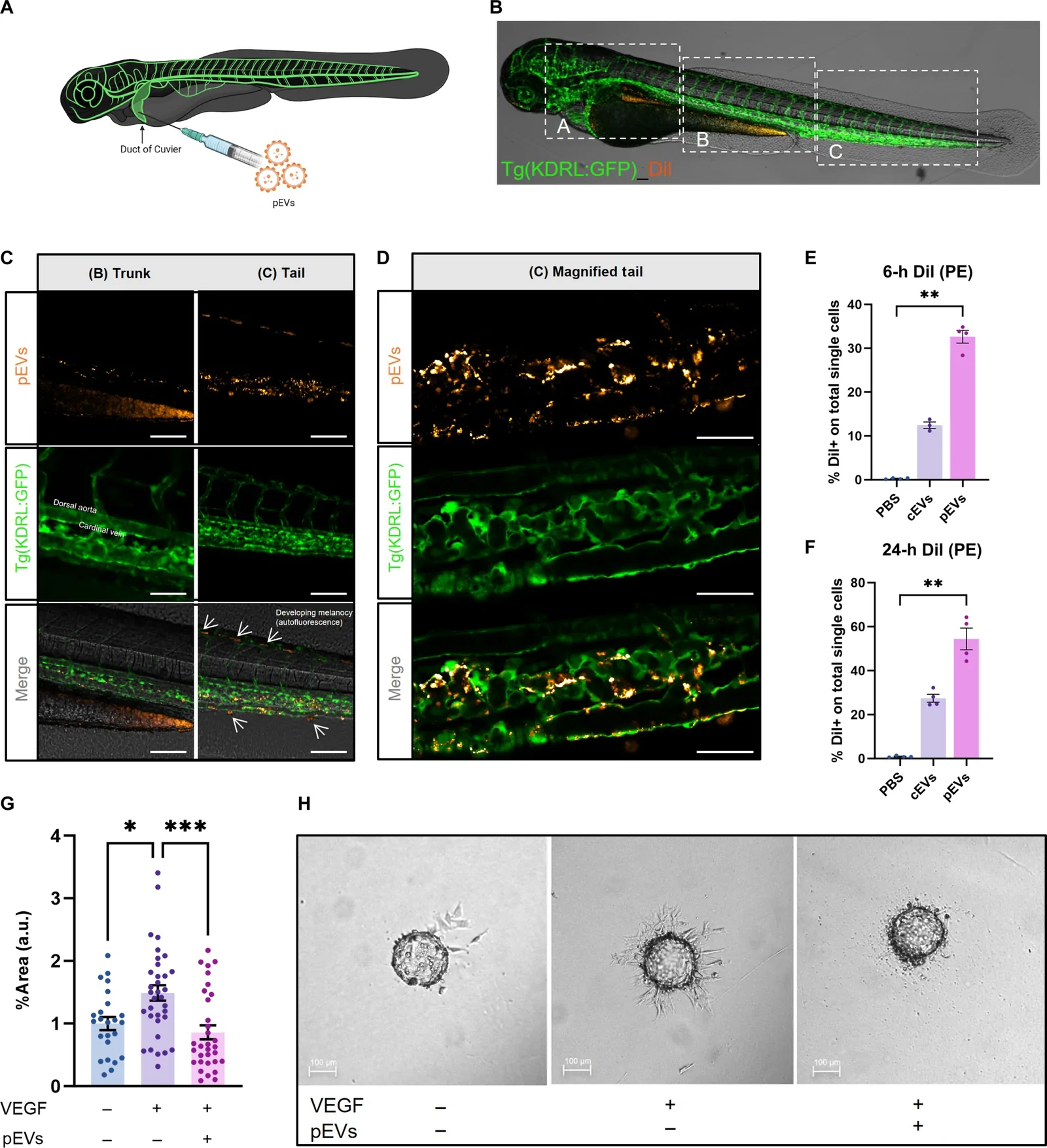
Extracellular vesicle (EV) biodistribution and uptake in endothelial cells. (A) Schematic representation of the experimental workflow: 1,1′-dioctadecyl-3,3,3′,3′-tetramethylindocarbocyanine perchlorate (DiI)-labeled pEVs were injected in the circulation through the duct of Cuvier in 48 h postfertilization (hpf) *Tg(KDRL:GFP)* zebrafish embryos. (B and C) Representative confocal images showing the biodistribution of DiI-pEVs at 24 h postinjection (hpi). Imaging areas are indicated in panel (B): A, yolk region; B, trunk region; and C, tail region. The endothelium is shown in green (green fluorescent protein [GFP]), and DiI-labeled pEVs are shown in orange. Scale bar, 100 μm. (D) Representative magnified confocal images of the tail region of DiI-pEV-injected zebrafish *Tg(KDRL:GFP)* embryos at 24 hpi. From top to bottom: orange channel (pEVs), green channel (endothelium), and merged image. Scale bar, 50 μm. (E and F) SVEC4-10 EV uptake in vitro by flow cytometry at 6 h (E) and 24 h (F). The percentage of PE (DiI)-positive cells on the total of single cells gated is represented. Data are representative of 2 independent experiments. Kruskal–Wallis test was applied. **P < 0.01. (G and H) Fibrin bead assay (FBA). (G) Quantification of FBA as percentage of area occupied by the sprouting around the beads and representative pictures (H). Data are representative of 14 images per condition from 3 independent experiments. Statistical differences were calculated by the Kruskal–Wallis test. *P < 0.05; ***P < 0.001. (H) Representative confocal images of FBA. Scale bar, 100 μm.

To investigate the biodistribution of pEVs, we performed an intravascular injection of DiI-prelabeled pEVs in transgenic *Tg(KDRL:GFP)* zebrafish embryos, expressing green fluorescent protein (GFP) in endothelial cells (schematic representation in Fig. 1A). Confocal imaging revealed specific accumulation of pEVs in the trunk and tail regions of the embryo, especially in the posterior cardinal vein (region B), and within the venous plexus of the tail (region C) (Fig. 1B and C). Indeed, pEV-injected embryos exhibited pronounced positivity for DiI signal colocalizing with endothelial GFP signal (Fig. 1C, with magnification in Fig. 1D), indicating the arrest and the uptake by endothelial cells of the venous compartment. Consistently, in vitro pEV uptake by murine endothelial cells (SVEC4-10), measured by gating DiI-positive cells at 6 and 24 h post-treatment, confirmed a significant increase in pEV uptake compared to control EVs (derived from nontreated MSCs) or PBS (Fig. 1E and F) at both time points. This differential uptake is independent of their number and size as cEVs and pEVs presented comparable concentration and diameters when analyzed by NTA (Fig. S1A to D). Overall, this suggests a preferential interaction of pEVs with the endothelium both in vivo and in vitro. Next, we moved to confirm the pEVs’ functional AA effect in vitro, using an FBA to mimic a 3D endothelial sprouting [17]. In the presence or absence of pro-angiogenic stimulation, we showed that, while, as expected, VEGF robustly induced sprouting, the addition of pEVs significantly inhibited this response (Fig. 1G and H), in agreement with our previous observations [10]. This confirms that pEVs not only target endothelial cells in vivo but also impair their angiogenic progress in vitro, reducing vessels sprouting in a process linked to matrix degradation.

### pEVs localize to tumor-associated vasculature and inhibit angiogenesis in zebrafish xenografts

**Fig. 2.**
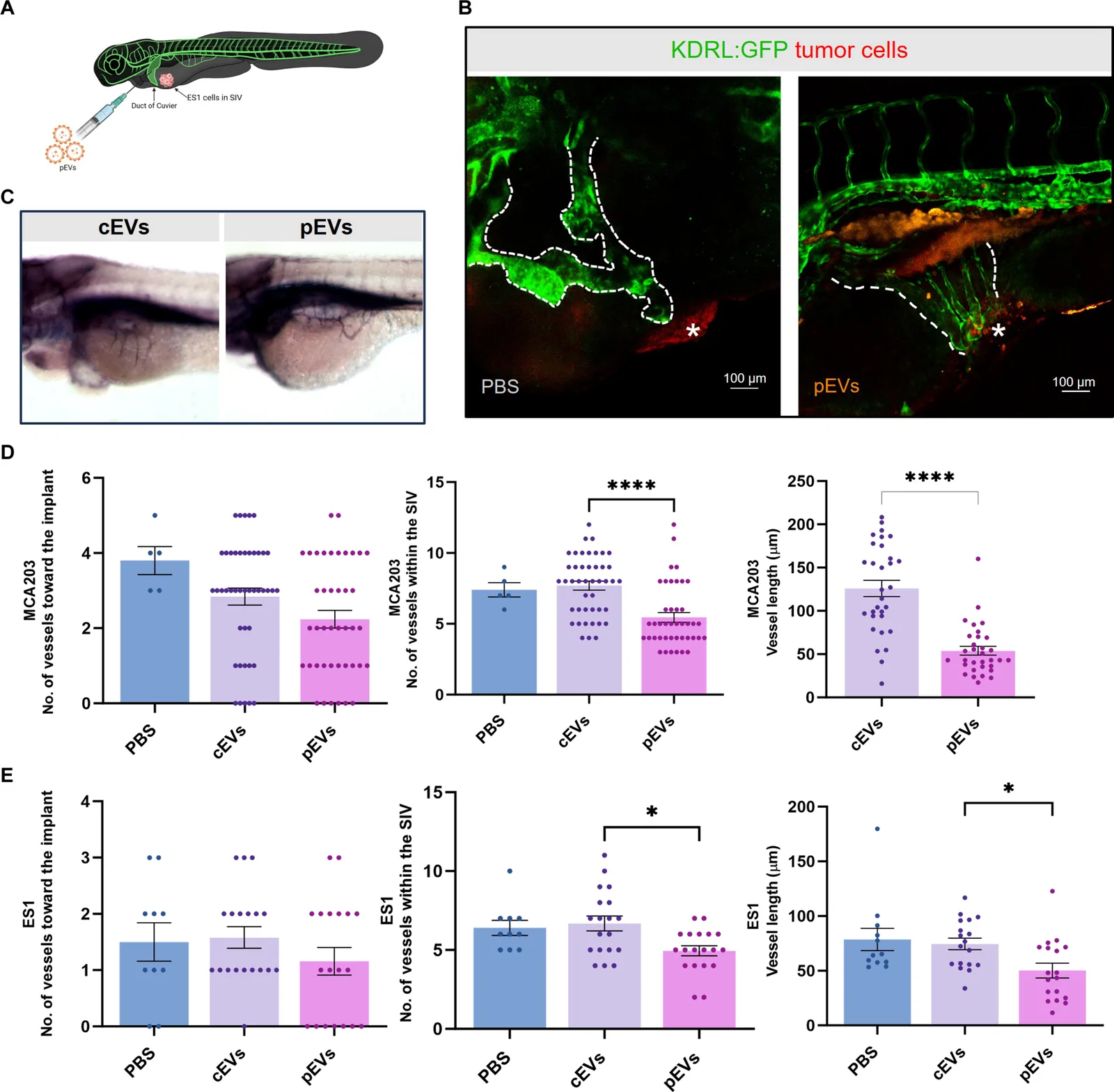
pEVs localize close to tumor-associated blood vessels (TABVs) and reduce angiogenesis in a zebrafish patient-derived (z-PDX) model. (A) Schematic representation of the z-PDX model. MCA203 or ES1 cells were injected into the perivitelline space of 48-hpf *Tg(KDRL:GFP)* zebrafish embryos. Phosphate-buffered saline (PBS), cEVs, and pEVs were subsequently injected in the circulation through the duct of Cuvier. (B) Confocal images of 24-hpi z-PDX MCA203 embryos (PBS-injected on the left and pEV-injected on the right). The vasculature is shown in green (green fluorescent protein [GFP]), tumor cells are in red (Qdot 655-stained MCA203), and 1,1′-dioctadecyl-3,3,3′,3′-tetramethylindocarbocyanine perchlorate (DiI)-stained pEVs are in orange. Asterisks indicate the site of tumor injection. Scale bar, 100 μm. (C) Representative images of alkaline phosphatase staining of cEV-injected and pEV-injected z-PDX at 24 hpi. Asterisks indicate the site of tumor injection. (D and E) Quantification of the number of vessels per embryo (left), the number of vessels within the subintestinal vein (SIV; center), and vessels’ length (right) in MCA203 (D) and ES1 (E) z-PDX embryos at 24 hpi. Each dot represents a single fish. Data are representative of *n* = 5 embryos for PBS, *n* = 44 embryos for cEVs, and *n* = 42 embryos for pEVs for MCA203 z-PDX (D) and *n* = 10 embryos for PBS, *n* = 19 embryos for cEVs, and *n* = 19 embryos for pEVs for ES1 z-PDX (E). Statistical differences were calculated by the Kruskal–Wallis test. *P < 0.05; ****P < 0.0001.

Building on these functional and biodistribution observations, we next sought to investigate whether pEVs retain their endothelial tropism and AA potential in a more complex and pathologically relevant context. To this end, we employed zebrafish xenograft models to mimic the tumor-associated vascular environment and evaluate the selective localization and therapeutic impact of pEVs within actively remodeling tumor vasculature. Two zebrafish xenograft models were established in parallel by injecting either murine sarcoma cells (MCA203) or human pediatric ES cells (ES1) into the perivitelline space of 48-hpf *Tg(KDRL:GFP)* embryos, in the proximity of the developing subintestinal vein (SIV) plexus (Fig. 2A) [19,20]. DiI-labeled pEVs were administered systemically via the duct of Cuvier after tumor implantation, and their biodistribution was assessed 24 h later using confocal microscopy. Although most of the pEVs were still found arrested in the posterior venous compartment, we observed an accumulation also in the proximity of tumor implants, colocalizing with endothelial cells at the tumor–vessel interface (Fig. 2B). Of note, PBS-injected controls show DiI-negligible endothelial association (Fig. 2B). Overall, this observation demonstrates the pEVs’ capacity to reach the actively sprouting endothelium within the TME. To assess the functional relevance of this interaction, we analyzed the developmental pattern of the SIVs in the zebrafish MCA203 xenograft model. Whereas cEVs control embryos exhibited aberrant growth of sprouting vessels from the SIV toward the tumor graft, embryos injected with pEVs showed a marked reduction in vessel outgrowth extending from the SIV toward the implant (Fig. 2C and D). Additionally, pEVs markedly decreased both the number of vessels within the SIV plexus and their cumulative length (Fig. 2D), corroborating the AA effect previously observed in vitro (Fig. 1G and H). Similar effects were reproduced in the zebrafish patient-derived xenograft (z-PDX) model using ES1 cells, where pEV administration significantly impaired vessel infiltration and angiogenic sprouting within the tumor-engrafted SIV region (Fig. 2E).

Overall, these findings further substantiate the ability of pEVs to accumulate near tumor-associated blood vessels (TABVs) and suggest that this selective localization is critical for their AA efficacy in vivo across distinct tumor models.

### TIMP-1 is a key mediator of pEV-induced AA activity

**Fig. 3.**
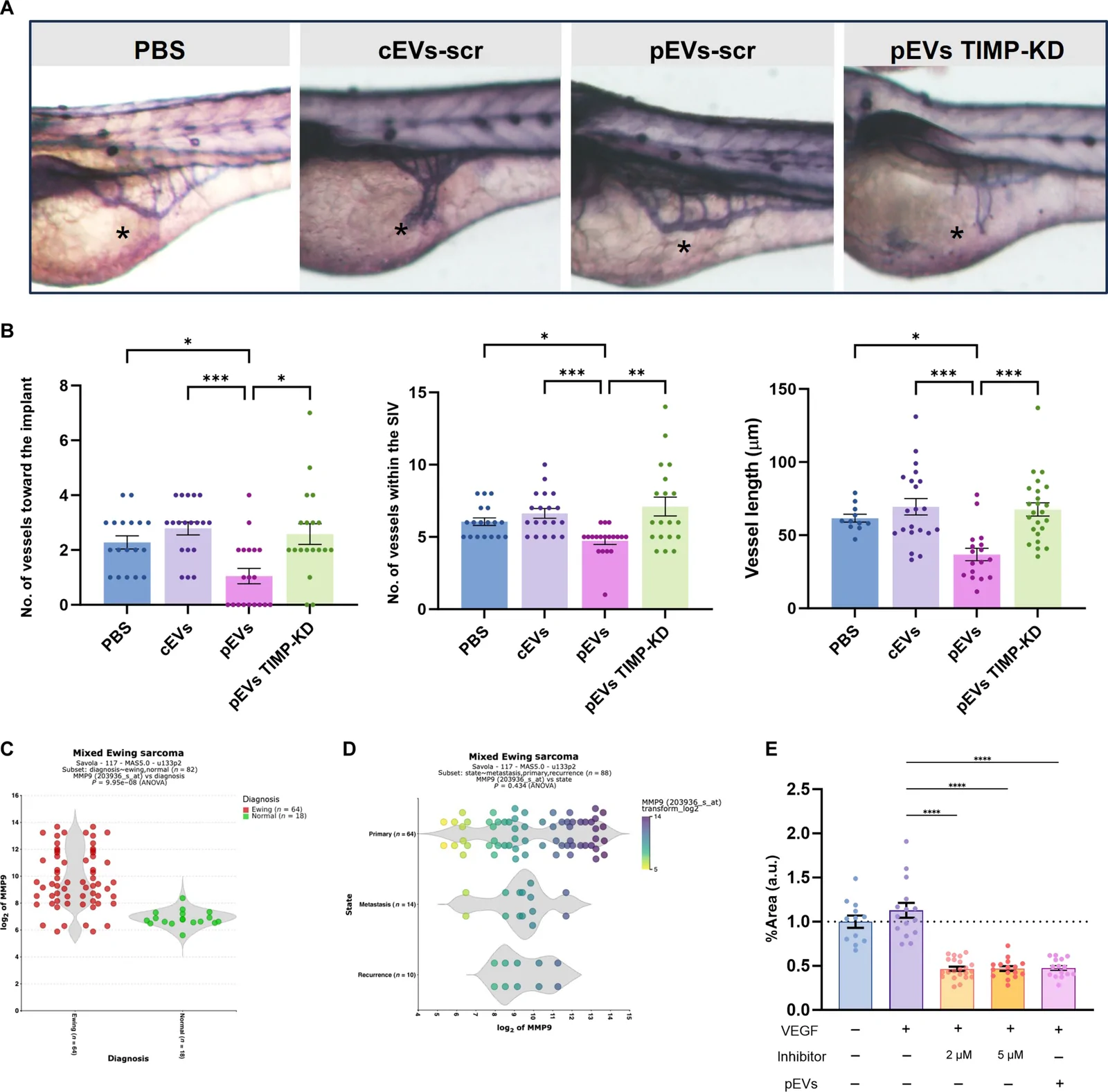
Tissue inhibitor of metalloproteinases-1 (TIMP-1) silencing reverts the anti-angiogenic (AA) pEV-mediated effect in a zebrafish patient-derived (z-PDX) Ewing sarcoma model. (A) Representative confocal images of alkaline phosphatase staining of z-PDX of Ewing sarcoma at 24 hpi. Asterisks indicate the site of tumor grafts. Treatments are indicated on the top of the images: phosphate-buffered saline (PBS), control EVs from unprimed cells treated with scramble siRNA (cEVs-scr), pEVs from primed MSCs treated with scramble siRNA (pEVs-scr), and TIMP-1-deficient pEVs obtained with TIMP-1 siRNA (pEVs TIMP-KD). (B) Quantification of number of vessels toward the implant (left), vessels within the subintestinal vein (SIV; center), and vessel length (toward the graph) (right) from alkaline phosphatase staining in z-PDX ES1. Each dot represents a single fish. Data are representative of *n* = 18 embryos for PBS, *n* = 19 embryos for cEVs, *n* = 19 embryos for pEVs, and *n* = 19 embryos for pEVs TIMP-KD from 2 independent experiments. Statistical differences were calculated by the Kruskal–Wallis test. *P < 0.05; **P < 0.01; ***P < 0.001. (C) Matrix metalloproteinase 9 (MMP9) expression in Ewing sarcoma patients. log_2_ of MMP9 expression is plotted against diagnosis (Ewing vs normal). Data are representative of *n* = 64 Ewing sarcoma patients and *n* = 18 normal subjects from the Savola cohort. Statistical differences were calculated by one-way analysis of variance (ANOVA). (D) MMP9 expression in Ewing sarcoma according to disease stage. log_2_ of MMP9 expression is plotted against the disease state (primary vs metastasis vs recurrence). Data are representative of *n* = 64 primary state, *n* = 14 metastasis state, and *n* = 10 recurrence state patients from the Savola cohort. Statistical differences were calculated by one-way ANOVA. (E) Fibrin bead assay (FBA) using different concentrations of an MMP inhibitor (2 and 5 μM) compared to pEVs. The percentage of area occupied by the sprouting around the beads normalized on the basal condition is represented. Single replicates are indicated, and mean ± SD is graphed. Data are representative of 2 independent experiments. One-way ANOVA with Sidak’s correction was applied after assessment of Gaussian distribution of data. ****P < 0.001.

Given the strong AA effect observed with pEVs, we next sought to define the molecular effector(s) responsible for this activity. TIMP-1, a tissue inhibitor of metalloproteinases known to regulate ECM remodeling and angiogenesis, was among the most abundant proteins detected in our EV preparations (Fig. S1A and B) [21]. To assess its functional relevance, we silenced TIMP-1 expression using an siRNA approach in pEV-producing MSCs [10]. Western blot analysis of the resulting EVs confirmed a marked reduction in TIMP-1 content in pEVs derived from primed MSCs transfected with TIMP-1-specific siRNA (pEVs TIMP-KD) (Fig. S2A and B). In contrast, pEVs from primed MSCs treated with scramble siRNA (pEVs-scr) retained TIMP-1 levels comparable to those observed in control EVs from unprimed cells (cEVs-scr and cEVs TIMP-1) (Fig. S2A and B). Notably, CD63 expression remained unchanged across all conditions, indicating that TIMP-1 knockdown was efficient and specific, without affecting EV integrity or identity (Fig. S2A and B). In vivo testing in our ES1 z-PDX model revealed that the AA activity of pEVs was abolished when TIMP-1 was depleted (Fig. 3A and B). The injection of pEVs-scr led to a marked reduction in TABVs as seen in Fig. 2E, while embryos treated with TIMP-1-deficient vesicles (pEVs TIMP-KD) exhibited dense vascular networks comparable to those of PBS- or cEV-treated controls (Fig. 3A and B). Quantitative analysis was performed to investigate the number of vessels oriented toward the tumor graft, the number of vessels within the SIV, and the overall vessel length (Fig. 3B). These data identify TIMP-1 as a major contributor of the AA activity of pEVs in an ES1 z-PDX model and support its potential therapeutic relevance in the modulation of TABV development. Importantly, transcriptomic analyses performed on human ES biopsies (R2 Genomics Analysis and Visualization Platform, http://r2.amc.nl) revealed a consistent up-regulation of several known TIMP-1 targets across primary tumors, metastatic lesions, and relapsed samples (*n* = 64) compared with normal tissues (*n* = 18). Among these, MMP9, a principal target of TIMP-1 and a key mediator of ECM remodeling, emerged as the most significantly overexpressed gene in ES samples (Fig. 3C). Interestingly, its expression was not scaling with disease progression (Fig. 3D), hinting toward an early and foundational role in ES outbreak and development. In addition, increased expression of other TIMP-1 target molecules as MMP13, MMP14, and MMP2 was observed in tumor samples, further supporting the presence of an enhanced proteolytic program linked to angiogenesis and tumor progression (Fig. S2C). Conversely, no substantial modulation was detected for other MMP family members that are not affected by TIMP-1, indicating a selective dysregulation of TIMP-1-sensitive targets (Fig. S2C) in ES pediatric biopsies. In this line, we have functionally confirmed in vitro that TIMP-1 inhibits endothelial sprouting by acting on MMP activity and in particular on MMP9 (Fig. 3E). Specifically, using FBA, we observed that pharmacological inhibition of MMP9 reduces endothelial sprouting, closely recapitulating the effect observed with pEVs carrying TIMP-1, its endogenous inhibitor. Together, this evidence not only corroborates the functional relevance of TIMP-1 in the regulation of tumor angiogenesis but also underscores the potential clinical value of targeting the TIMP-1/MMP axis in ES.

### PMMA-based NPs enable TIMP-1 encapsulation and delivery

**Fig. 4.**
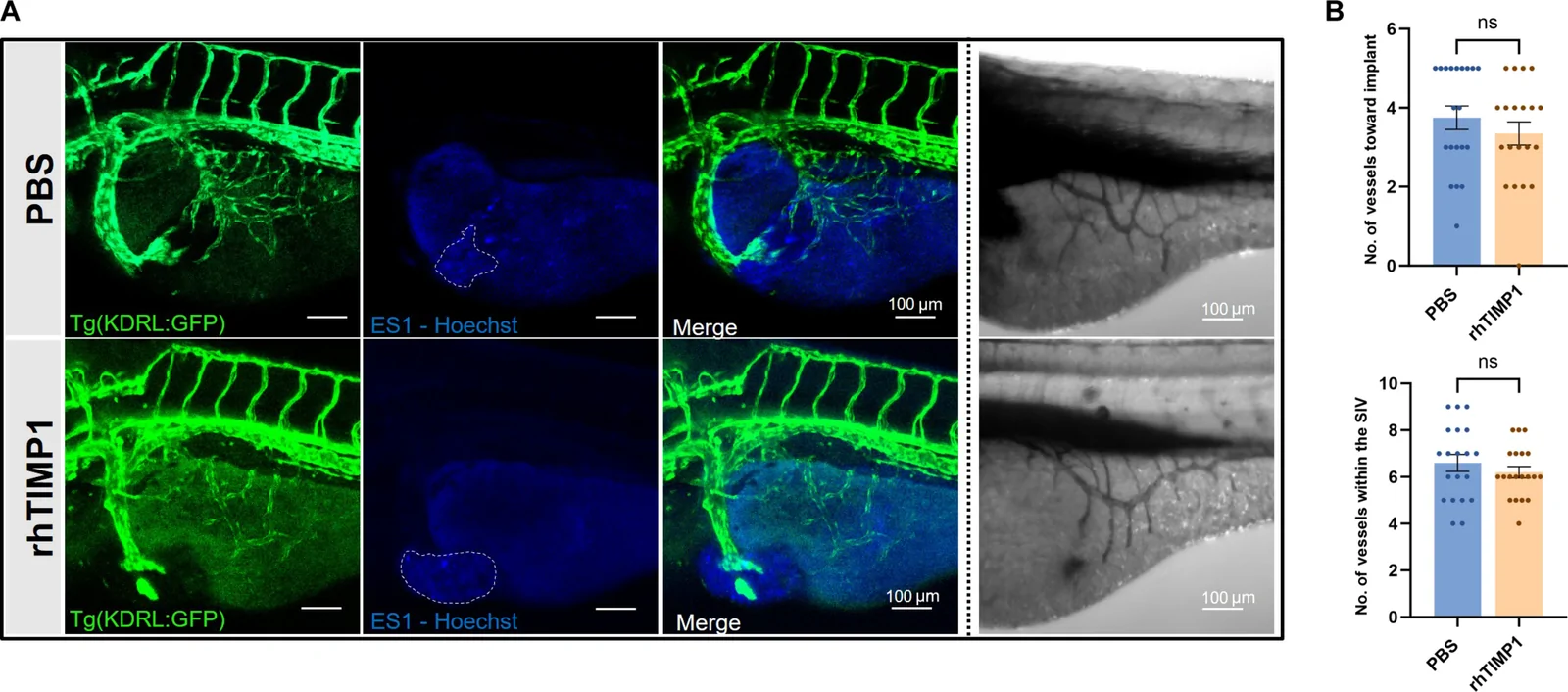
Soluble recombinant human TIMP-1 (rhTIMP1) does not impair tumor-associated blood vessel (TABV) formation in a zebrafish patient-derived xenograft (z-PDX) Ewing sarcoma model. (A) Representative confocal images of *Tg(KDRL:GFP)* z-PDX ES1 embryos at 48 hpf injected with phosphate-buffered saline (PBS; top) or rhTIMP1 (bottom). From left: endothelial cell signal (green), ES1 tumor cell signal (blue), merged signal, and alkaline phosphatase staining of z-PDX ES1 embryos at 24 hpi. The area occupied by tumor cells is contoured by a white-dotted line. Scale bar, 100 μm. (B) Quantification of the number of vessels growing toward the tumor implant (top) and the number of vessels within the subintestinal vein region (bottom) in z-PDX ES1 embryos injected with PBS or rhTIMP1. Each dot represents a single fish. Data are representative of *n* = 20 embryos for PBS (top) and *n* = 20 embryos for rhTIMP1 (bottom), from 1 independent experiment. Statistical differences were calculated by the Mann–Whitney test; ns, *P* > 0.05.

Prompted by the relevance of TIMP-1 in mediating the AA effect of pEVs and by the clinical importance suggested by patient database analysis, we decided to investigate whether the recombinant protein alone (rhTIMP1) would be able to fully replace pEV administration in vivo. Thus, we injected rhTIMP1 into the z-PDX ES model (ES1 cells) (Fig. 4). Strikingly, the administration of rhTIMP1 did not inhibit tumor-associated angiogenesis (Fig. 4A and B), despite a fully active enzymatic activity (Fig. S2D), suggesting a specific advantage of TIMP-1 carried by EVs in mediating the AA effect in vivo. However, the employment of EVs in clinical practice is unfortunately highly limited due to their poor developability in terms of production and batch-to-batch reproducibility [22].

**Fig. 5.**
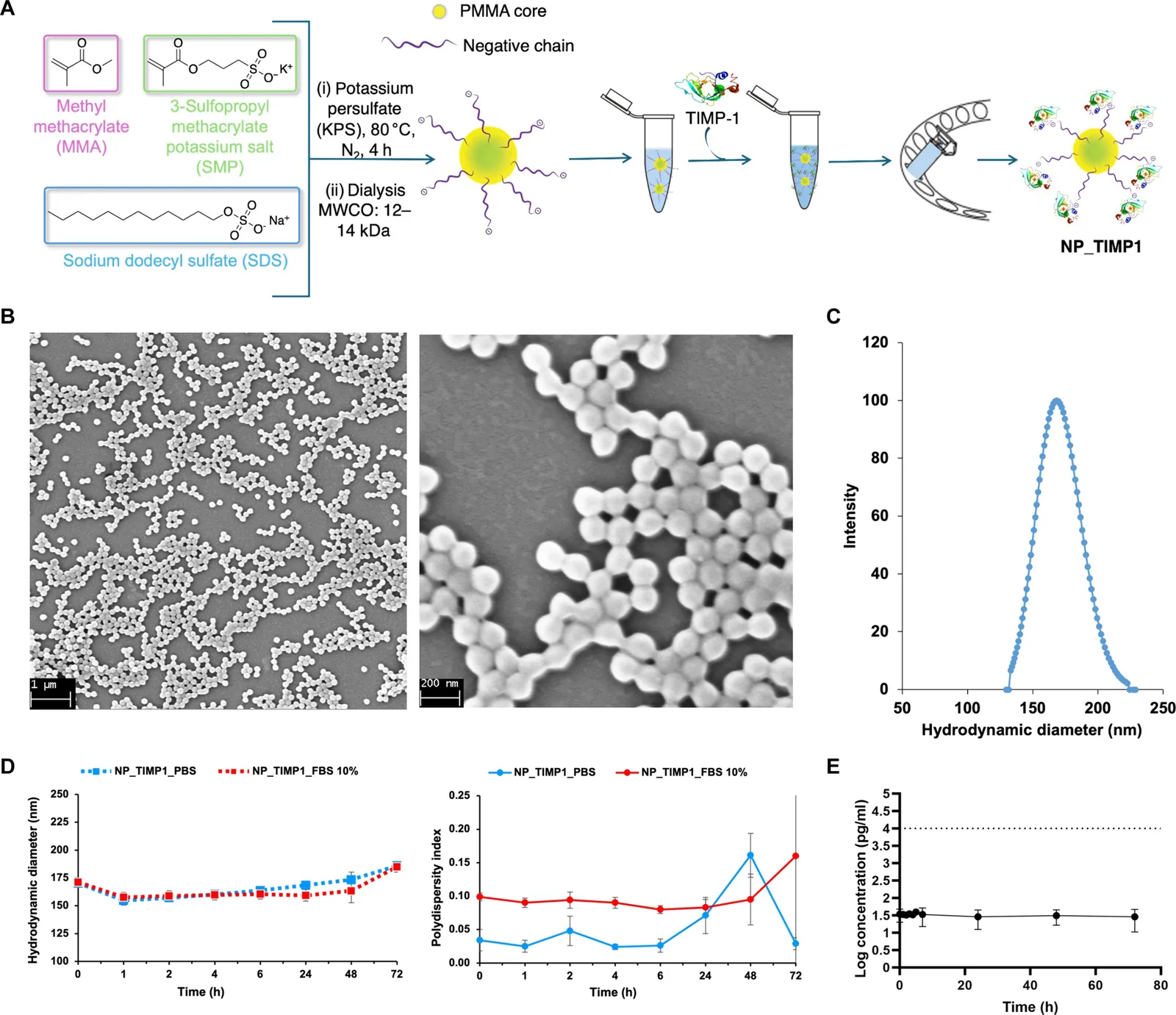
NP_TIMP1 synthesis and characterization. (A) Nanoparticle synthesis and schematic representation. (B) NP_TIMP1 scanning electron microscopy (SEM) analysis (scale bars 1 μM and 200 nm) at 2 different magnifications, e.g., 20,000× and 100,000×. (C) Representative example of NP_TIMP1 hydrodynamic diameter distribution. (D) From left: NP_TIMP1 size trend (hydrodynamic diameter) in PBS (dotted blue line) and phosphate-buffered saline (PBS) + 10% fetal bovine serum (FBS) (red dotted line) at 37 °C, over 3 d; NP_TIMP1 polydispersity trend in PBS (blue line) and PBS + 10% FBS (red line) at 37 °C, over 3 d. Data are representative of 3 independent experiments. (E) Stability of TIMP-1 loading in nanoparticles. The concentration of TIMP-1 (expressed in log of pg/ml) is plotted against time (h). Data are representative of 2 independent experiments.

In this line, strong efforts have been made to replace them with nanostructures recapitulating vesicles’ size, concentration, and content. Here, NPs have gained a central position in contributing to the development of novel delivery strategies. Indeed, NPs constitute a robust drug-delivery platform, allowing precision therapeutics with control of release kinetics and loading efficiency [23]. Negatively charged core–shell PMMA NPs were synthesized via an emulsion copolymerization process (Fig. 5A), yielding uniform particles with an average hydrodynamic diameter of approximately 160 nm (Fig. 5B and C) and a *ζ*-potential of −54 mV. Subsequently, TIMP-1 was loaded onto the NP surface through electrostatic interactions, leading to the formation of NP_TIMP1 particles with an average hydrodynamic diameter of 167 nm and a *ζ*-potential of −22 mV. The pronounced decrease in the magnitude of the negative surface charge (−22 mV vs −54 mV) clearly indicated the successful binding of the positively charged TIMP-1 onto the NPs’ shell. Nevertheless, the retention of a slightly negative *ζ*-potential suggests that the NPs preserved their electrostatic repulsion, thus maintaining colloidal stability. Morphological characterization by SEM revealed that the NPs exhibited a spherical shape and were uniformly distributed, as shown in Fig. 5B. The measured dry diameter was approximately 135 nm, in good agreement with the hydrodynamic size determined by DLS. The colloidal stability of NP_TIMP1 was further evaluated at 37 °C for 72 h in PBS and in PBS supplemented with 10% FBS at pH 7.4 (Fig. 5D). DLS measurements revealed no appreciable changes in hydrodynamic diameter or polydispersity index under either condition, and no signs of aggregation or precipitation were observed over time. These results demonstrate that TIMP-1 loading did not alter the structural integrity or dispersion properties of the NPs. In addition, we performed a time-course analysis of TIMP-1 retention on NP_TIMP1 under physiological conditions. As shown in Fig. 5E, we did not observe a statistically significant release of TIMP-1 over time, even at extended time points (up to 72 h). The measured levels of TIMP-1 in the supernatant remained low and relatively stable, indicating that the protein remains largely associated with the NP surface. This evidence supports the stability of the electrostatic loading strategy under the tested conditions, suggesting that TIMP-1 is retained in a NP-bound configuration rather than being rapidly released in solution. This highlights its potential suitability for in vitro and in vivo applications.

### Human endothelial cell function is impaired upon exposure to NP_TIMP1

**Fig. 6.**
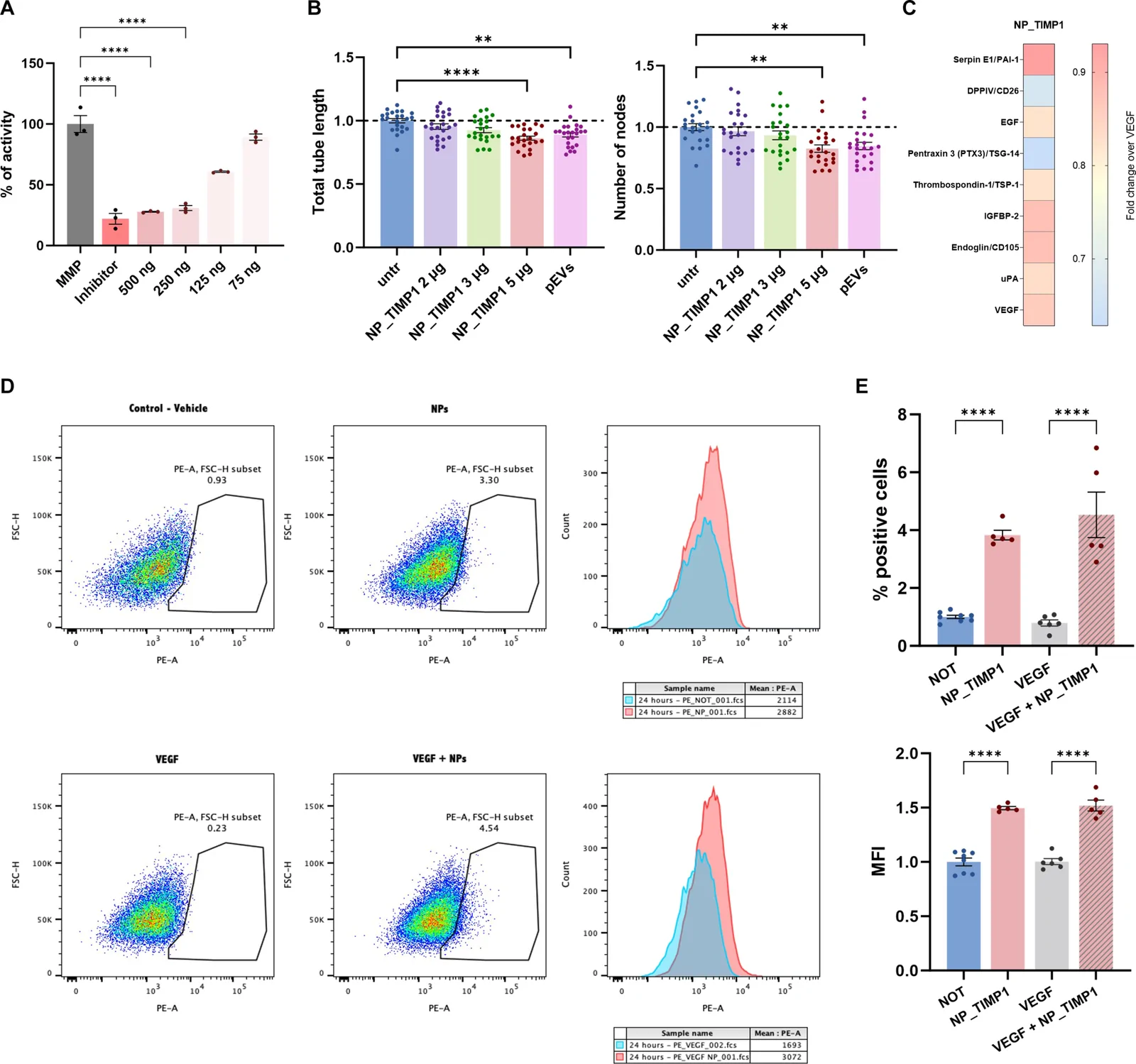
NP_TIMP1 retains anti-angiogenic (AA) and uptake properties comparable to those of pEVs. (A) Dose-dependent inhibition of matrix metalloproteinase (MMP) enzymatic activity by NP_TIMP1, as assessed by fluorescent zymography, compared with a reference small-molecule MMP inhibitor. The percentage of enzymatic activity (% of activity) per treatment is plotted. Data are representative of 3 independent experiments. Statistical differences were calculated by one-way analysis of variance (ANOVA). ****P < 0.0001. (B) Quantification of total tube length (left) and branching nodes (right) from tube formation assay in human umbilical vein endothelial cells (HUVECs), upon treatment with NP_TIMP1 at increasing concentration of tissue inhibitor of metalloproteinases-1 (TIMP-1; 2 to 5 μg). Data are representative of 25 images per condition from 3 independent experiments. Statistical differences were calculated by the Kruskal–Wallis test. **P < 0.01; ****P < 0.0001. (C) Proteome profiler array of NP_TIMP1-loaded nanoparticles. Heatmap representation of angiogenesis-related protein expression in NP_TIMP1-treated HUVECs relative to vascular endothelial growth factor (VEGF)-stimulated controls. VEGF-treated samples were used as reference condition (fold change = 1). Values below 1 indicate reduced expression upon NP_TIMP1 treatment, with lower values (blue tones) corresponding to stronger down-regulation. (D and E) NP_TIMP1 internalization in HUVECs by flow cytometry. (D) Representative flow cytometry plots illustrating NP_TIMP1 internalization under basal and VEGF-stimulated conditions. (E) Quantification of percentage of positive cells (top) and mean fluorescence intensity (MFI) (bottom) from flow cytometry analysis. Data are representative of 2 independent experiments. Statistical differences were calculated by one-way ANOVA. ****P < 0.0001.

As treatment with soluble rhTIMP1 failed to recapitulate pEVs’ AA effect, we next sought to determine whether, instead, human TIMP-1-loaded NPs (NP_TIMP1) could succeed in reiterating pEVs’ molecular and functional properties. As a first step, we evaluated the ability of NP_TIMP1 to inhibit MMP enzymatic activity, a key downstream target of TIMP-1. Using a fluorescent zymography-based assay, we observed a clear dose-dependent suppression of MMP activity following NP_TIMP1 treatment (Fig. 6A). These data confirm that TIMP-1 maintains its biological activity upon NP loading and it is functionally available to modulate proteolytic activity. We next tested whether NP_TIMP1 could impair endothelial angiogenic behavior. In a tubule formation assay using human endothelial cells (HUVECs, ATCC), treatment with 5 μg of NP_TIMP1 resulted in a substantial reduction of total tube length and branching complexity (Fig. 6B). Based on these results, 5 μg was selected as the optimal dose for subsequent in vitro experiments. To further assess the impact of NP_TIMP1 at the molecular level, we complemented our functional analysis with a proteome profiler assay evaluating angiogenesis-related proteins (Fig. 6C). This analysis revealed that NP_TIMP1 modulated the VEGF-induced angiogenic protein profile. For instance, NP_TIMP1 lowered the levels of pro-angiogenic DPPIV/CD26 and pentraxin 3 (PTX3), supporting their ability to attenuate endothelial angiogenic activation beyond the direct inhibition of MMPs (Fig. 6C). Furthermore, given the preferential endothelial interaction of pEVs (Fig. 1), we tested whether NP_TIMP1 shares this feature. Flow cytometry analysis in HUVECs confirmed NP internalization, even under VEGF stimulation (Fig. 6D). Both the percentage of positive cells and the mean fluorescence intensity were increased in comparison to those of untreated cells, indicating an efficient endothelial uptake of NPs (Fig. 6E). Overall, these findings demonstrate that NP_TIMP1 not only retains the protease-inhibitory function of TIMP-1 but also efficiently interacts with endothelial cells and inhibit angiogenic behavior in vitro, thereby functionally mirroring the effects of TIMP-1-enriched pEVs through a fully synthetic, scalable, and modular system.

### NP_TIMP1 inhibits endothelial sprouting in an all-human 3D angiogenesis model

**Fig. 7.**
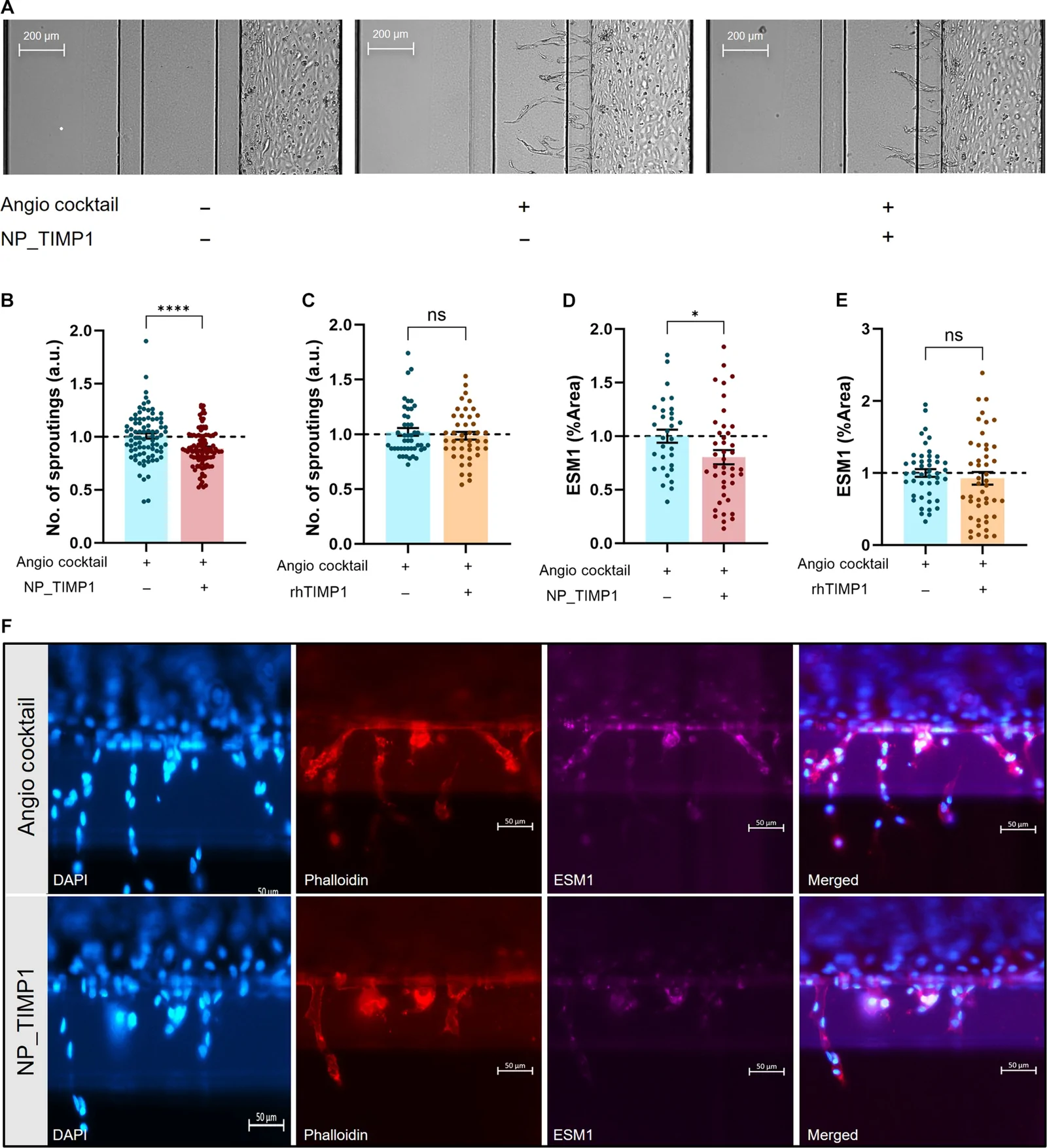
NP_TIMP1 reduces sprouting in an in vitro microfluidic model. (A) Representative live imaging of human umbilical vein endothelial cells (HUVECs) grown into 3-dimensional (3D) tubular structures in the Mimetas OrganoPlate system 3 d post-treatment with the angiogenic cocktail (Angio cocktail) and/or NP_TIMP1. Scale bar, 200 μm. (B and C) Quantification of sprouting from live imaging of HUVEC 3D cultures 3 d post-treatment with the angiogenic cocktail (Angio cocktail) and/or NP_TIMP1 (B) or rhTIMP1 (C). (B) The number of sproutings (a.u.) normalized on the mean of the Angio cocktail group is represented. Data are representative of *n* = 96 (Angio cocktail) and *n* = 90 (NP_TIMP1) images from 6 independent experiments. Statistical differences were calculated by the Mann–Whitney test; ****P < 0.0001. (C) The number of sproutings (a.u.) normalized on the mean of the Angio cocktail group is represented. Data are representative of *n* = 42 (Angio cocktail) and *n* = 48 (rhTIMP1) images from 3 independent experiments. Statistical differences were calculated by the Mann–Whitney test; ns, *P* > 0.05. (D and E) Quantification of the percentage of the ESM1-positive area in cells treated with the angiogenic cocktail (Angio cocktail) and/or NP_TIMP1 (D) or rhTIMP1 (E). (D) The percentage of area positive for ESM1 signal (%Area) normalized on the mean of the Angio cocktail is represented. Data are representative of *n* = 42 (Angio cocktail) and *n* = 31 (NP_TIMP1) images from 2 independent experiments. Statistical differences were calculated by the Mann–Whitney test. *P < 0.05. (E) The percentage of area positive for ESM1 signal (%Area) normalized on the mean of the Angio cocktail group is represented. Data are representative of *n* = 46 (Angio cocktail) and *n* = 45 (rhTIMP1) images from 2 independent experiments. Statistical differences were calculated by the Mann–Whitney test. ns, *P* > 0.05. (F) Representative images of the confocal microscopy of ESM1 staining comparing the angiogenic cocktail only (top) and NP_TIMP1 treatment (bottom). From left: 4′,6-diamidino-2-phenylindole (DAPI; blue), phalloidin (red), ESM1 (magenta), and merged. Scale bar, 50 μm.

Having confirmed that NP_TIMP1 retains both the enzymatic inhibitory activity of TIMP-1 and the capacity to preferentially target endothelial cells, we next aimed to further characterize their AA properties using a physiologically relevant in vitro model. To better mimic the dynamic, spatially organized process of angiogenic sprouting, we employed the OrganoReady Collagen 3-lane 64 (Mimetas, Oegstgeest, the Netherlands), a microfluidic platform that enables 3D co-culture under perfused, multi-stimuli conditions (schematic representation in Fig. S3A). In this system, endothelial cells were exposed to a defined angiogenic cocktail to promote sprouting (Angio cocktail; see Materials and Methods), while NP_TIMP1 were administered directly via the perfusion channel to simulate the delivery in vivo. Live imaging revealed extensive endothelial sprouting into the central perfusion lane after stimulation with the angiogenic cocktail (Fig. 7A). This response was markedly attenuated by NP_TIMP1 (Fig. 7A and B), while NP_empty did not exert any effect (Fig. S3B). To note, rhTIMP1 alone could not mimic the AA effect of NP_TIMP1, even at a 10-fold concentration, as previously observed in vivo (Fig. 7C and Fig. S3C). Consistent with the reduced endothelial sprouting observed upon NP_TIMP1 treatment, we further reported by immunofluorescence a lower presence of leading tip cells, marked by ESM1, a VEGF-inducible marker commonly associated with tip-cell identity (Fig. 7D to F). Again, rhTIMP1 did not demonstrate any significant effect (Fig. 7E). Altogether, these results indicate that NP_TIMP1 impacts key features of the angiogenic process in vitro, as shown by reduced endothelial sprouting and lower detection of ESM1-positive tip cells within a 3D microfluidic environment that closely mimics physiological vascular architecture.

**Fig. 8.**
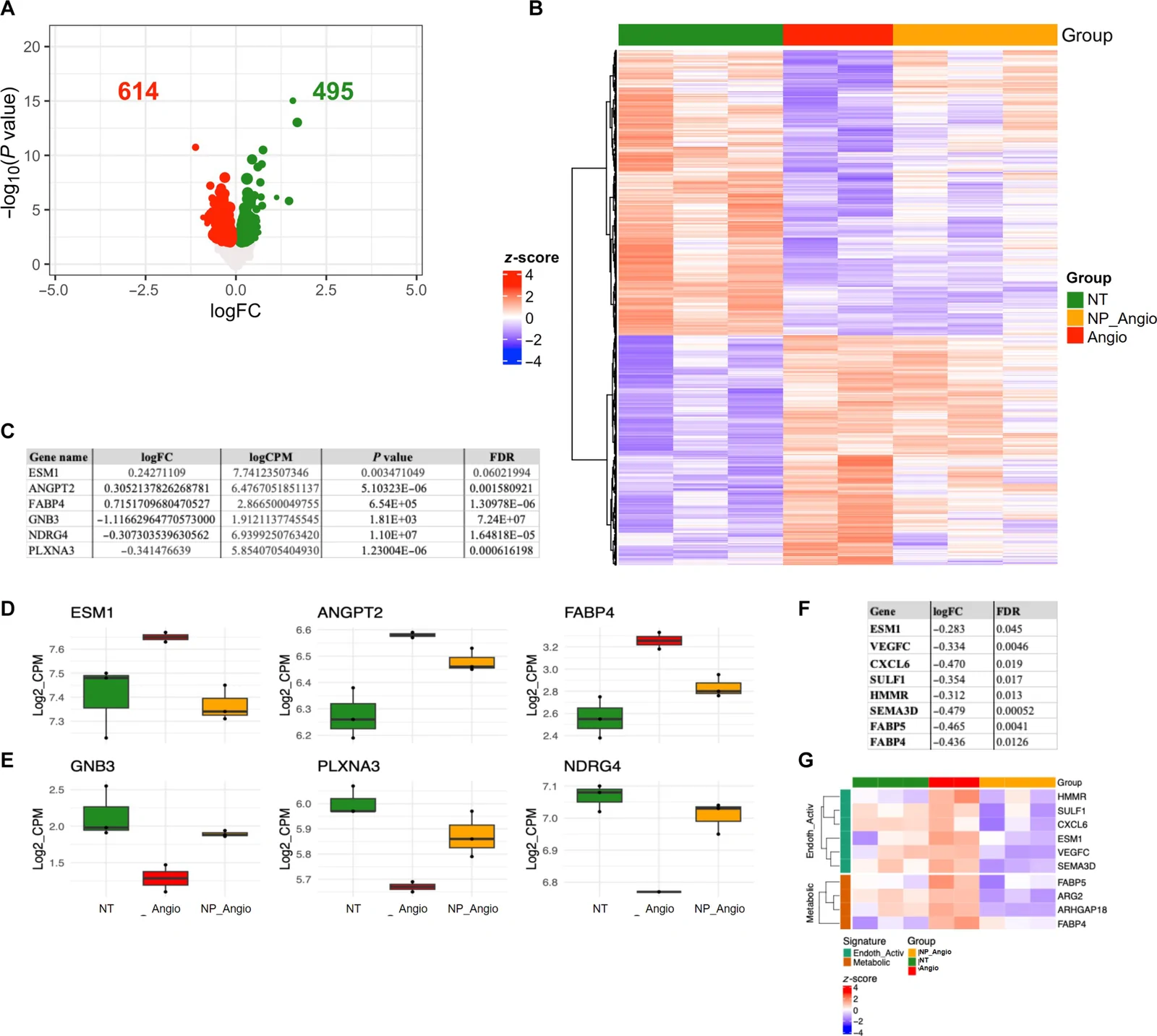
Bulk RNA sequencing (RNA-seq) analysis of endothelial cells cultured in the Mimetas microfluidic system. (A) Volcano plot showing differentially expressed genes (DEGs) in angiogenic-cocktail-stimulated endothelial cells (Angio, red) compared with nontreated controls (NT, green). (B) Heatmap of Angio DEGs across the other groups. (C to E) Summary table and relative graphs of selected DEGs between Angio and NT conditions. Data are representative of *n* = 15 chips for negative control (NT, green), *n* = 16 chips for angiogenic cocktail (Angio, red), and *n* = 16 chips treated with NP_TIMP1 in combination with the angiogenic cocktail (NP_Angio) from 3 independent experiments. (F) Focused analysis of NP_Angio versus Angio cells showing attenuation of selected tip-cell/sprouting- and matrix-remodeling-associated genes. (G) Heatmap representing the *z*-score of selected endothelial activation and metabolic activation signatures from NP_Angio DEGs across conditions. Data are representative of *n* = 15 chips for negative control (NT, green), *n* = 16 chips for angiogenic cocktail (Angio, red), and *n* = 16 chips for NP_TIMP1 (NP_Angio) from 3 independent experiments.

To further support the impact of NP_TIMP1 on endothelial behavior in the 3D microfluidic system, we performed bulk RNA-seq analysis on HUVECs exposed to the Angio cocktail in the presence or absence of NP_TIMP1. First, we confirmed that the angiogenic cocktail efficiently induced a transcriptional angiogenic response. Differential expression analysis of treated cells versus nontreated controls revealed a broad transcriptional remodeling, with 495 up-regulated and 614 down-regulated genes (Fig. 8A). Consistently, the global heatmap showed a clear separation between nontreated (NT, green) and angiogenic-cocktail-stimulated samples (Angio, red), confirming the induction of a distinct pro-angiogenic endothelial program (Fig. 8B). In this setting, the treatment with NP_TIMP1 (NP_Angio, yellow) of cells stimulated with the angiogenic cocktail affects the sprouting phenotype, resembling the one of the unstimulated conditions. In detail, the angiogenic cocktail significantly increased the expression of ESM1, together with ANGPT2 and FABP4, proteins involved in the vascular remodeling and the metabolic adaptation of activated endothelial cells, as summarized in the table in Fig. 8C. The overexpression of these genes was blunted by the NP_TIMP1 treatment (Fig. 8D). Consistently, the angiogenic-cocktail-driven inhibition of GNB3, PLXNA3, and NDRG4 was blocked by NP treatment (Fig. 8E). Together, these data demonstrate that the pro-angiogenic stimulation induces an active endothelial signaling in the microfluidic model, characterized by activation of tip-cell-associated, vascular remodeling, and metabolic reprogramming features. Gene Ontology analysis further supported this interpretation. Angio versus NT DEGs were enriched for biological processes related to proliferation, cytoskeletal remodeling, phosphorylation, and cell–substrate adhesion, consistent with an active angiogenic endothelial state (Fig. S3D). Conversely, NP_Angio versus Angio DEGs were enriched for ECM- and vascular-lumen-associated terms, suggesting that NP_TIMP1 preferentially remodels the matrix-associated and vascular-compartment features of the angiogenic response (Fig. S3E). Indeed, NP_TIMP1 attenuated selected components of the angiogenic-driven program, with a particular impact on genes linked to tip-cell specification, motility/matrix interaction, and metabolic reprogramming (Fig. 8F and G). Among these, the reduction of ESM1 is especially relevant, as it is consistent with the decreased ESM1-positive tip-cell area observed by immunofluorescence in the same microfluidic setting (Fig. 7D to F). Overall, these data indicate that NP_TIMP1 remodels the angiogenic-driven endothelial transcriptional program, limiting the acquisition of a tip-cell-like and matrix-remodeling phenotype.

### In vivo anti-angiogenic activity of NP_TIMP1 recapitulates the effects of pEVs

**Fig. 9.**
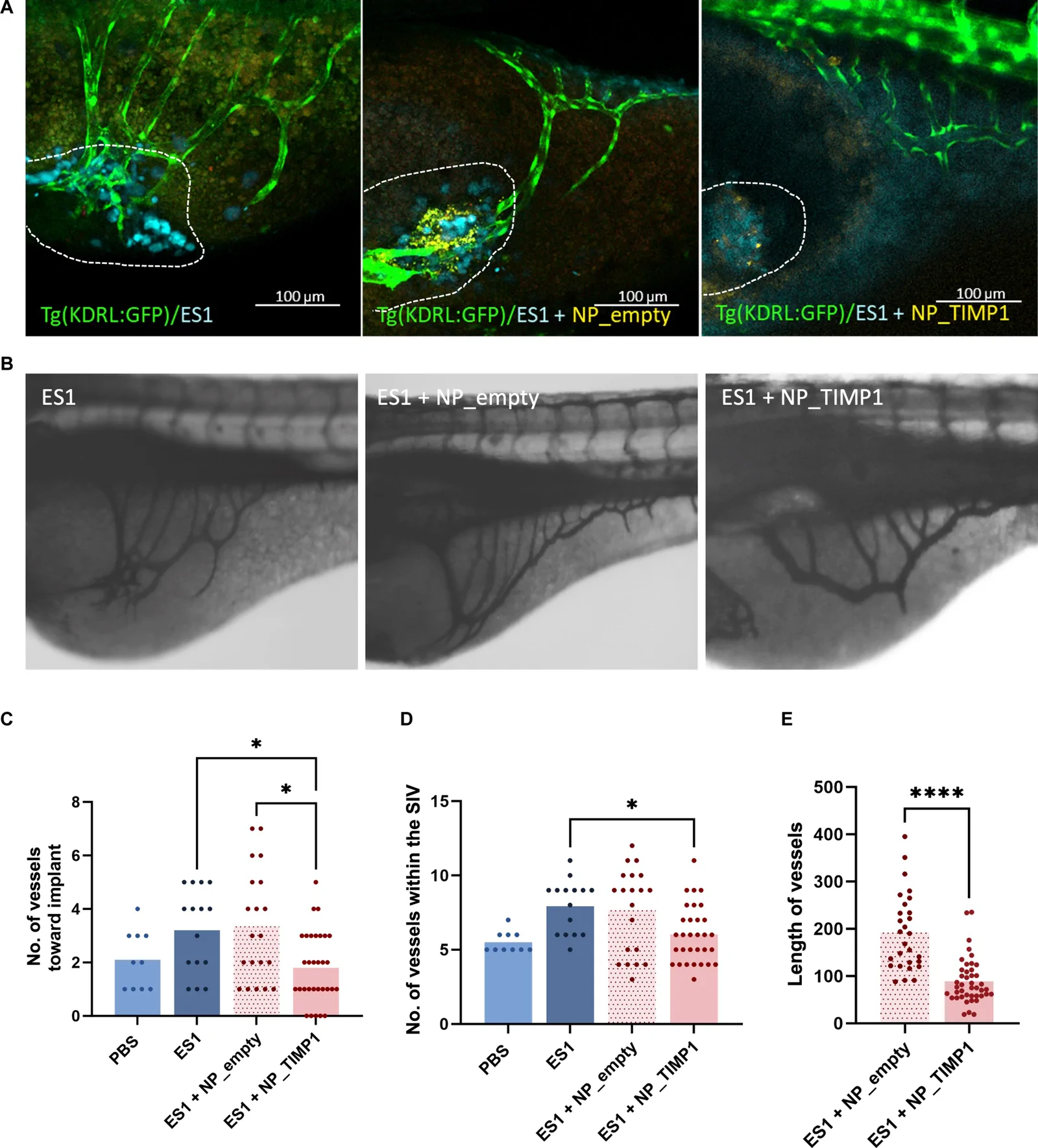
NP_TIMP1 localizes close to tumor-associated blood vessels (TABVs) and reduces angiogenesis in a zebrafish patient-derived xenograft (z-PDX) Ewing sarcoma model. (A) Representative confocal images of *Tg(KDRL:GFP)* z-PDX ES1 embryos at 24 hpi injected with phosphate-buffered saline (PBS; left), NP_empty (center), and NP_TIMP1 (right). Endothelial cells are shown in green, ES1 cells in blue, and nanoparticles (NPs) in yellow. Scale bar, 100 μm. (B) Representative images of alkaline phosphatase staining of z-PDX ES1 embryos at 24 hpi. ES1 + PBS (left), ES1 + NP_empty (center), and ES1 + NP_TIMP1 (right). (C to E) Quantification of the number of vessels toward the implant (C), number of vessels within the subintestinal vein (SIV) (D), and length of vessels (μm) (E). Each dot represents a single fish. Data are representative of *n* = 15 embryos for ES1, *n* = 20 embryos for ES1 + NP_empty, and *n* = 31 embryos for ES1 + NP_TIMP1 from 2 independent experiments. Statistical differences were calculated by one-way analysis of variance (ANOVA). *P < 0.05; ****P < 0.0001.

After confirming the successful biochemical encapsulation of TIMP-1 into PMMA-based NPs and demonstrating their AA effects on primary human endothelial cells in vitro, we next investigated their efficacy in vivo in our z-PDX ES model. The in vivo administration of NP_TIMP1 did not show any signs of systemic toxicity, such as morphological abnormalities or reduced survival (data not shown). Given their safety profile, we characterized their biodistribution and localization in vivo. We initially confirmed that NP_TIMP1 preferentially colocalized within the venous compartment of the trunk and tail regions, in a manner similar to that observed for NP_empty at 1 hpi (Fig. S4A and B), resembling the distribution pattern previously reported for pEVs (Fig. 1). Next, we coinjected human ES1 cells with either NP_empty or NP_TIMP1 and quantified angiogenesis 1 d postimplantation. Confocal imaging revealed that the presence of NP_TIMP1 in the graft region markedly reduced vascular density and sprouting compared to PBS- and NP_empty-injected embryos where the tumor-mediated angiogenesis was still evident (Fig. 9A). Alkaline phosphatase staining confirmed the AA effect of TIMP-1-loaded NPs in vivo, as the embryos injected with NP_TIMP1 displayed a visibly reduced vessel network surrounding the tumor implant compared to control groups (Fig. 9B). Quantitative morphometric analysis revealed that NP_TIMP1 treatment significantly decreased the number of vessels extending toward the tumor (Fig. 9C), the number of intratumoral vessels within the SIV plexus (Fig. 9D), and the cumulative vessel length (Fig. 9E), closely mirroring the vascular phenotype previously observed upon treatment with TIMP-1-enriched pEVs. These findings provide in vivo evidence that TIMP-1-loaded PMMA NPs effectively reproduce the AA effects of pEVs in a clinically relevant z-PDX model, highlighting their potential as a standardized and bioactive AA therapeutic platform.

## Discussion

The limited and transient efficacy of current AA therapies underscores the urgent need for alternative strategies capable of durably modulating tumor vascularization. The redundancy of pro-angiogenic signaling and the rapid activation of compensatory mechanisms, particularly in highly plastic tumors such as pediatric ES, have constrained the long-term success of VEGF/VEGFR blockade. In this context, EVs have emerged as powerful mediators of stromal communication, capable of delivering complex molecular cues that regulate angiogenesis and tissue remodeling. However, their therapeutic translation remains hampered by major pharmaceutical and regulatory challenges, including heterogeneity, scalability, and storage instability.

Building upon our previous identification of pro-inflammatory primed MSC-derived EVs (pEVs) as potent AA agents, we demonstrated here that their efficacy critically depends on the metalloproteinase inhibitor TIMP-1. By combining mechanistic insight with nanotechnology, we engineered polymeric NPs that replicate the biological function of TIMP-1-enriched EVs in a fully synthetic and scalable form. Our PMMA-based nanoparticles (NP_TIMP1) encapsulate recombinant human TIMP-1 while preserving its enzymatic inhibitory activity, leading to efficient suppression of metalloproteinase (MMP) function and endothelial sprouting in both 2D and 3D models. To note, our data show that NP_TIMP1 is efficiently internalized by human endothelial cells in vitro and preferentially localizes to venous regions in vivo. Importantly, within these vascular niches, NP_TIMP1 reduced endothelial sprouting and tip-cell formation. Together, these findings establish NP_TIMP1 as an EV-mimetic nanotherapeutic capable of recapitulating the spatiotemporal regulation of angiogenesis exerted by stromal vesicles. Although our in vitro models do not fully reproduce the complexity of the TME, they were instrumental in comparing NP_TIMP1 with pEVs and in dissecting the functional contribution of TIMP-1. Our results indicate that TIMP-1 likely exerts its AA function by modulating ECM remodeling during endothelial sprouting, consistent with its known biological role. In this context, the particulate presentation of TIMP-1, either within EVs or NP carriers, emerged as a key determinant of its AA efficacy. Soluble TIMP-1, despite retaining enzymatic activity, failed to reproduce the effects of NP_TIMP1 or TIMP-1-enriched EVs (pEVs). This finding hints toward the potential importance of spatial confinement and surface anchoring of TIMP-1 at the nanoscale as critical determinants of its biological function. Moving from this piece of evidence, we are tempted to speculate that such a configuration likely enhances the local concentration of TIMP-1 near the endothelial surface and ECM, stabilizing its bioactive conformation and facilitating interactions with proteolytic substrates. The NP surface might act as a bioengineering interface that transforms a diffusible protein into a spatially constrained signaling cue, an effect that natural EVs achieve through membrane presentation. This putative mechanism highlights how nanoscale architecture can directly influence protein bioactivity and tissue targeting.

From a translational standpoint, TIMP-1-based NPs (NP_TIMP1) might represent a complementary and orthogonal approach to conventional anti-VEGF therapies. Rather than targeting pro-angiogenic growth factors, NP_TIMP1 modulates the ECM remodeling that sustains vascular expansion. Importantly, in ES, our transcriptomic analyses revealed the up-regulation of several TIMP-1-sensitive proteases, including MMP9, MMP13, and MMP14, emphasizing a proteolytic program that may be therapeutically exploitable. Molecular analyses further showed that NP_TIMP1 attenuates selected VEGF-induced angiogenesis-associated mediators, including DPPIV/CD26 and PTX3 [24–27], and reshapes the VEGF-driven endothelial program in the 3D endothelial culture system (OrganoReady, Mimetas). Further mechanistic dissection of these pathways will highlight their importance as valuable targets for tumors. By acting at this ECM-remodeling level, NP_TIMP1 could be integrated with existing regimens to achieve synergistic vascular normalization or AA effects. Together, these findings support the concept that NP_TIMP1 can functionally replace the AA activity of complex pEVs through a more defined and scalable NP-based system. To support the therapeutic relevance of this approach, it was therefore essential to validate NP_TIMP1 activity in an in vivo model able to capture tumor-associated vascular remodeling in a patient-derived context. The zebrafish z-PDX model provided a powerful in vivo platform for high-resolution visualization of tumor-associated angiogenesis. Using this model, we showed that NP_TIMP1 localizes to the vascular compartment and reduces tumor-associated sprouting in a patient-derived context. These findings support the relevance of this approach as a first functional validation of the AA activity of NP_TIMP1. Future mammalian studies will be required to assess therapeutic efficacy, biodistribution, safety, and long-term effects. However, these studies should be guided by a deeper characterization of the vascular programs active in ES PDX models, which remain largely unexplored. This knowledge will be essential to define clinically relevant vascular readouts and to guide the subsequent preclinical evaluation of this therapeutic strategy.

## Conclusion

This study provides a proof of concept for the rational design of EV-inspired nanotherapeutics that translate stromal biology into engineered platforms (Fig. S5). The NP_TIMP1 system unites the precision and reproducibility of synthetic materials with the functional complexity of biological signals. Beyond angiogenesis, this approach opens opportunities for modular bioengineering of vesicle-mimetic systems tailored to specific microenvironmental pathways. Future work will aim to define the pharmacokinetics, therapeutic window, and safety profile of NP_TIMP1 in mammalian models, as well as to explore combination strategies with chemoradiotherapy and other vascular modulators. More broadly, the same vesicle-mimetic design principle could be adapted to other bioactive proteins or regulatory cues involved in inflammation, fibrosis, or tissue regeneration, paving the way for a new generation of nanotherapeutics that integrate biological insight with material precision.

## Data Availability

All data produced in the present study are available upon reasonable request to the corresponding author. The sequencing data were deposited in Gene Expression Omnibus (GEO; https://www.ncbi.nlm.nih.gov/geo/) (GEO trackID: GSE334430).
