# Supplementary material for: Bioinspired TIMP-1 Nanoparticles Mimic Extracellular Vesicle Anti-angiogenic Activity in Ewing Sarcoma
Source: Biomater Res. 2026 Sep 22;30:0408. doi: 10.34133/bmr.0408 (PMC13594418; doi:10.34133/bmr.0408)
Supplement: Supplementary file 1 [file bmr.0408.f1.docx]

Supplementary Materials for

**Bioinspired TIMP-1 Nanoparticles Mimic Extracellular Vesicle Anti-angiogenic Activity in Ewing Sarcoma**

Cristina Liboni, Martina Boaron *et al.*

*Corresponding author. Email: roberta.angioni@unipd.it

**This file includes:**

Figs. S1 to S5

**Fig. S1 relative to Fig. 1 and 3.**

**Characterization of EVs. A.** Representative WB of control EVs (cEVs) and primed EVs (pEVs). Molecular weights of proteins are indicated on the right. **B.** WB quantification. Protein expression levels were normalized on CD63 as housekeeping protein. The relative expression levels on cEVs are represented. Data are representative of 3 independent experiments. One sample t test after assessment of normality distribution (Shapiro-Wilk test) was applied. **C.** Representative Nanosight profiles of control EVs (cEVs) and primed EVs (pEVs). **D.** Nanosight quantification of EVs size. Data are representative of n=5 (cEVs) and n=6 (pEVs) different preparations analyzed in 3 independent experiments.

**A**

TIMP1 d

CD63

28kDa

60-30kDa

scr

cEVs

scr

pEVs

cEVs

TIMP-KD

pEVs

TIMP-KD

**B**

**D**

**C**

**Fig. S2 relative to Fig. 3 and 4.**

**Characterization of TIMP-1 silenced EVs. A.** WB quantification. Protein expression levels were normalized on CD63 as housekeeping protein. Data are representative of 3 independent experiments. **B.** Representative WB of scramble control EVs (scr cEVs), scramble primed (scr pEVs), TIMP1-KD control (cEVs TIMP-KD) and TIMP1-KD primed (pEVs TIMP-KD). Molecular weights of proteins are indicated on the right. **C.** MMPs expression in Ewing sarcoma patients (R2: Genomics Analysis and Visualization Platform - http://r2.amc.nl). Log2 of MMPs expression is plotted against diagnosis (Ewing vs normal). Data are representative of n=64 Ewing sarcoma patients and n=18 normal subjects from Savola cohort. Statistical differences were calculated by One-way ANOVA. **D.** Dose-dependent inhibition of MMP enzymatic activity by rhTIMP1, as assessed by fluorescent zymography, compared with a reference small-molecule MMP inhibitor. The percentage of enzymatic activity (% of activity) per condition is represented. Data are representative of 4 independent experiments. Statistical differences were calculated by one-way ANOVA. ****p<0.0001

**A**

**B**

**C**

**D**

**E**

**Fig. S3 relative to Fig.7-8**.

**3D-validation of TIMP1_NP effect on Mimetas plate. A.** Schematic representation of the Mimetas OrganoPlate^Ⓡ^ system, which enables HUVEC cells to grow into a 3D tubular structure against an ECM (seeded in the orange channel). The blue channel gets perfused with an angiogenic cocktail, which promotes sprouting, while cells are directly perfused with NP_TIMP1. **B.** Quantification of sproutings from live imaging of HUVEC 3D cultures 3 days post treatment with NP_empty. The number of sproutings (a.u.) normalized on the mean of the angiogenic cocktail (angio cocktail, see Material and Methods) group is represented. Data are representative of n=17 (angio cocktail) and n=25 (NP_empty) images per condition from 2 independent experiments. Statistical differences were calculated by Mann-Whitney test; ns p>0,05. **C.** Quantification of sprouting from live imaging of HUVEC 3D cultures 3 days post treatment with rhTIMP1 at different concentrations (1-fold and 10-fold the concentration employed in nanoparticle formulations). The number of sprouting (a.u.) normalized on the mean of the angio cocktail group is represented. Data are representative of n=24 (angio cocktail), n=23 (rhTIMP1 1X) and n=12 (rhTIMP1 10X) images from 3 independent experiments. Statistical differences were calculated by Kruskal-Wallis test; ns p>0,05. **D.** Gene Onthology analysis of Angio versus NT DEGs representing the top 15 BP (biological processes). **E.** Gene Onthology analysis of NP_Angio versus Angio DEGs representing the top 15 CC (cellular components). For graphs in D and E, log10 (p-adjusted) is plotted on the x-axis, BP or CC are plotted on the y-axis. The gene count is coded by the size of the bubble while the gene ratio is color-coded (legend on the right).

**Fig. S4 relative to Fig.9**.

**Immunofluorescence representative pictures of in vivo z-PDX experiments. A-B.** Representative confocal images of NP_empty (A) and NP_TIMP1 (B) biodistribution in *Tg (KDRL:GFP)* zebrafish embryos at 1hpi. From the top: head, trunk and tail portions are represented. From left to right: endothelium signal (green), NPs signal (orange) and merged signal. Scale bar, 100µm.

**Fig. S5**

**Graphical abstract.** Bioengineered TIMP-1 nanoparticles (NP_TIMP1) are proposed as an anti-angiogenic platform for tumoral vascular targeting. In vitro assays and zebrafish patient-derived xenograft (z-PDX) models of Ewing sarcoma demonstrate that NP_TIMP1 preserve TIMP-1 bioactivity and enhance endothelial targeting.
